# Severe neurodegeneration in brains of transgenic rats producing human tau prions

**DOI:** 10.1007/s00401-024-02771-5

**Published:** 2024-08-20

**Authors:** Jacob Ayers, T. Peter Lopez, Ian T. Steele, Abby Oehler, Rigo Roman-Albarran, Elisa Cleveland, Alex Chong, George A. Carlson, Carlo Condello, Stanley B. Prusiner

**Affiliations:** 1 Institute for Neurodegenerative Diseases, Weill Institute for Neurosciences, University of California, San Francisco, CA 94158 USA; 2 Department of Neurology, Weill Institute for Neurosciences, University of California, San Francisco, CA 94158 USA; 3 Department of Biochemistry and Biophysics, University of California, San Francisco, CA 94158 USA

**Keywords:** Tauopathy, MAPT, Hyperphosphorylation, Misfolding, Prions, Transgenic rat

## Abstract

Both wild-type and mutant tau proteins can misfold into prions and self-propagate in the central nervous system of animals and people. To extend the work of others, we investigated the molecular basis of tau prion–mediated neurodegeneration in transgenic (Tg) rats expressing mutant human tau (P301S); this line of Tg rats is denoted Tg12099. We used the rat *Prnp* promoter to drive the overexpression of mutant tau (P301S) in the human 0N4R isoform. In Tg12099(+/+) rats homozygous for the transgene, ubiquitous expression of mutant human tau resulted in the progressive accumulation of phosphorylated tau inclusions, including silver-positive tangles in the frontal cortices and limbic system. Signs of central nervous system dysfunction were found in terminal Tg12099(+/+) rats exhibiting severe neurodegeneration and profound atrophy of the amygdala and piriform cortex. The greatest increases in tau prion activity were found in the corticolimbic structures. In contrast to the homozygous Tg12099(+/+) rats, we found lower levels of mutant tau in the hemizygous rats, resulting in few neuropathologic changes up to 2 years of age. Notably, these hemizygous rats could be infected by intracerebral inoculation with recombinant tau fibrils or precipitated tau prions from the brain homogenates of sick, aged homozygous Tg12099(+/+) rats. Our studies argue that the regional propagation of tau prions and neurodegeneration in the Tg12099 rats resembles that found in human primary tauopathies. These findings seem likely to advance our understanding of human tauopathies and may lead to effective therapeutics for Alzheimer’s disease and other tau prion disorders.

**Supplementary Information:**

The online version contains supplementary material available at 10.1007/s00401-024-02771-5.

## Introduction

Tau is encoded by the *MAPT* gene and is highly expressed in neurons where it promotes axonal microtubule assembly [19, 51, 77]. In addition to amyloid-β plaques, neurofibrillary tangles (NFTs) composed of the misfolded tau protein [2, 12, 32, 37, 50, 80] are the neuropathological hallmarks of Alzheimer’s disease (AD). While tau was known to be involved in AD pathology, its role in primary tauopathies and “frontotemporal lobar degeneration-tau” (FTLD-tau) cases was not known until monogenic missense and splice-site mutations were discovered in the *MAPT* gene [42, 64, 72]. Growing evidence argues that the pathological spread of misfolded tau in AD, Down syndrome, FTLD-tau, and other tauopathies is due to the self-templating and propagation of tau prions in the central nervous system (CNS) [5, 10, 21, 25, 44, 66, 78, 81]. Strains of tau prions manifest distinct biochemical and neuropathological properties that are retained in serial passages in cell culture and in animal models [17, 49, 66]. Recent advances in cryo–electron microscopy have revealed that these distinct strain properties are due to variations in the conformation of the misfolded tau protein. Distinct conformations of tau have been observed in brain samples from patients with AD, chronic traumatic encephalopathy, progressive supranuclear palsy (PSP), corticobasal degeneration, and other tauopathies [26–28, 70, 83].

Studies using transgenic (Tg) mouse models of tauopathy have helped establish that pathological tau is involved in tau prion propagation, disease pathogenesis, and neuronal dysfunction [1, 4, 22, 53, 55, 67, 82]. The widely used TgPS19 and Tg2541 mouse models express MAPT*P301S, an aggressive early onset FTLD-tau mutation, and develop high levels of tau prions in the brainstem, cerebellum, and midbrain and lower levels in forebrain structures like the cortex and hippocampus [1, 47, 79, 82]. Another widely used mouse model (rTg4510) exhibits corticolimbic-specific tau pathology [67], but the bigenic transgene configuration disrupts six genes, including *Fgf14* [30, 35]. This gene is critical for neuronal signaling and synapse function [74], so its disruption may confound interpretation of tauopathy phenotypes and neurodegeneration in the rTg4510 model. New rodent models in which we can investigate tau prion formation in the forebrain, NFT formation, and neurodegeneration are needed to understand the disease mechanisms of primary tauopathies.

Rats are a favorable alternative to mice as rodent models of neurodegeneration because they have an extensive brain network consisting of almost 200 million neurons—nearly three-fold more than a mouse brain [41]. The rat’s complex CNS has facilitated sophisticated neuropsychiatric behavioral modeling and may prove to be a better system in which to investigate the functional consequences of neurodegeneration. Previously, we developed a novel vector to overexpress the rat prion protein (PrP) in the Tg rat brain [56]. We found that intracerebral inoculation of a single rat-adapted PrP strain into two different Tg(PrP) rat lines caused accelerated disease kinetics with neuropathological spread and neurodegeneration in the corticolimbic system of one line and in the hindbrain and brainstem of the other line [56]. These earlier studies using Tg rats with modified prion disease phenotypes allowed us to determine which Tg rat brain regions are permissive to prion replication and vulnerable to damage [56]. We leveraged our knowledge from these previous studies of rat PrP disease to investigate tau prion propagation in relation to neurodegeneration in the Tg rat brain.

In the work reported here, we describe Tg rats that overexpress the MAPT*P301S mutation (Tg12099 rats) throughout the CNS to examine tau prion spread. Homozygous Tg12099(+/+) rats developed progressive abnormal phosphorylation of human tau (hTau) and mature NFTs in the forebrain and limbic system in contrast to phenotypically healthy hemizygous Tg12099(+/−) rats. Extensive focal neurodegeneration of the corticolimbic system, resembling FTLD-tau, coincided with tau pathology in the Tg12099(+/+) rats. To determine if the corticolimbic system contained tau prions, we longitudinally tested various brain regions from both Tg12099(+/+) and Tg12099(+/−) rats using a quantitative cellular assay that detects tau prion levels in brain lysates. We found that tissue from the corticolimbic systems of Tg12099(+/+) rats had the highest levels of tau prion activity. Taken together, homozygous Tg12099(+/+) rats expressing the MAPT*P301S mutation had increased tau prion propagation in the corticolimbic system, formation of NFTs, and neurodegeneration restricted to the forebrain region. This pathology was found as early as 6 months of age and rapidly progressed to 1 year of age. The phenotypes observed in the Tg12099(+/+) rat more accurately depict FTLD-tau in human patients than current animal models. This novel rat model should facilitate future investigations of the disease mechanisms of tau prion propagation and concurrent neurodegeneration. Although the hemizygous Tg12099(+/−) rat did not spontaneously accumulate tau prions, exogenous administration of recombinant tau fibrils and brain homogenates from aged Tg12099(+/+) rats induced tau prion accumulation in the forebrain. Together, our data support the Tg12099 rat as a potential resource for testing molecules to treat tauopathy in humans.

## Methods

### Husbandry and clinical assessment of rats

Rats were maintained in an AAALAC-accredited facility in accordance with the *Guide for the Care and Use of Laboratory Animals*. All animal procedures were approved by the University of California, San Francisco Institutional Animal Care and Use Committee. All rats were examined at least once per day for any abnormal clinical sign(s) using a systematic neurological protocol that included assessment of ambulation, lack or presence of hind leg clasping, presence or absence of ataxia, and testing for the righting reflex. Rats displaying two or more clinical signs were identified; if their clinical condition was unchanged or deteriorated within 24 h, then they were euthanized to define the incubation period in these studies. For each animal study, an equal number of male and female rats were used.

### Construction of RaPrnp-MAPT*P301S Tg rats

The 0N4R isoform of human *MAPT* complementary DNA (cDNA) used in this study was a gift from Sue-Ann Mok (University of Alberta). We modified this cDNA to incorporate the MAPT*P301S mutation by site-directed mutagenesis. MAPT*P301S cDNA was amplified by polymerase chain reaction (PCR) and cloned into the RaPrnp vector via In-Fusion cloning at the *Xho*I site. The resulting RaPrnp-MAPT*P301S construct was linearized by *Not*I digestion and microinjected into the pronuclei of Sprague–Dawley rat zygotes to establish the Tg12099 line. For complete transgenesis methods in Sprague–Dawley rats, refer to Lopez et al. [56]. To determine the genotype of Tg12099 rats, genomic DNA (gDNA) from ear punches was purified and applied to a real-time PCR reaction. Primer sequences used to modify or amplify gene products are listed in Supplementary Table S1.

### RaPrnp-MAPT*P301S copy number detection in Tg12099 rats

Copy number of RaPrnp-MAPT*P301S in Tg12099 rats was determined by droplet digital PCR (ddPCR) as described in Lopez et al. [56]. Briefly, purified gDNA from Tg12099(+/−) rats was digested with *Mse*I at 37 °C for 1 h. Digested gDNA containing *Prnp*-FAM (target; Bio-Rad #10042958) and *Rpp30*-Hex (reference; Bio-Rad #10042961) probes were applied to ddPCR reactions and analyzed on a QX200 droplet reader (Bio-Rad). QuantaSoft software (Bio-Rad) was used to calculate copy number variation by comparing the concentration of *Prnp* target (*A*) to the concentration of the *Rpp30* (*B*) reference loci in gDNA samples. In the equation, *NB* refers to two copies of *Rpp30* in the rat genome:

$$\text{CNV}=\frac{A}{B}\times \text{NB}$$

### Determining the RaPrnp-MAPT*P301S genomic insertion site by targeted locus amplification

Two primer sets were designed to the RaPrnp-MAPT*P301S transgene: one set to the *RaPrnp* promoter and the other set to the human *MAPT* open reading frame (Supplementary Table S1). Viable rat spleen cells were processed for targeted locus amplification (TLA) [23] and combined with primer sets in individual TLA reactions. PCR products were purified to generate a library via a Nextera DNA Flex kit (Illumina) and sequenced on an Illumina sequencer. Reads were mapped using BWA-SW [54], version 0.7.15-r1140, with a setting of bwasw-b 7. The sequencing reads of the RaPrnp-MAPT*P301S transgene were aligned to the rat genome, assembly rn6. TLA in Tg12099 rats was conducted by Cergentis as a paid service.

### Generation and DNA sequencing of a rat Mapt knockout line

In collaboration with a contract research organization (Cyagen), we used transcription activator-like effector nucleases (TALENs) to generate a rat *Mapt* (0/0) allele (known as Cy23). gDNA isolated from ear punches from Cy23 rats were purified and used as template for PCR reactions using rat *Mapt* Exon 2 primers (Table S1). Amplified PCR products were column purified and were sequenced by Single Primer Extension using 5′ or 3′ rat *Mapt* Exon 2 primers through Sequetech as a paid service. Chromatograms of wild-type (WT; +/+) Exon 2 sequence were used to determine a two-base pair deletion (GA) in +/0 (Cy23) rats.

### Polyacrylamide gel electrophoresis (PAGE) genotyping analysis of Cy23 rats

To screen F2 progeny for rat Mapt knockouts (0/0), a PAGE-based genotyping approach was adapted from Zhu et.al. [84]. Briefly, PCR reactions were prepared with 100 ng/µL of gDNA from WT (+/+) control or F2 progeny along with PAGE Mapt genotyping primers (Table S1) that amplify the TALEN-targeted region of rat *Mapt* Exon 2. PCR conditions used: an initial preincubation step at 98 °C for 30 s followed by 98 °C for 10 s, 67.3 °C for 20 s, and 72 °C for 15 s, repeated for 30 cycles. The final extension was at 72 °C for 10 min and cooled to room temperature (unmixed samples). Once PCR reactions were completed, the (+/+) control sample was mixed at a 1:1 volume with each F2 PCR product. Mixed samples were denatured at 95 °C for 5 min and cooled to room temperature to allow for annealing and the formation of DNA heteroduplexes and homoduplexes. Unmixed or mixed PCR samples were then combined with 1× Hi-Density TBE buffer (Novex, LC6678) and loaded onto a 20% PAGE gel (Novex, EC63155BOX). Electrophoresis was performed until the bromophenol blue dye–front progressed to ~ 75% the length of the gel. PAGE gels were then immersed in running buffer containing 1× SYBR gold gel stain (Invitrogen, S11494) for 10 min, and DNA bands were imaged on a Chemidoc Imaging system (Bio-Rad).

### Validation of Cy23 rats by tau Western blotting and immunohistochemistry (IHC)

We bred the Cy23 line to homozygosity and determined Mapt protein levels in brain homogenates using antibodies targeted to different domains of the full-length tau protein. Data from Western blots of brain homogenates and IHC on fixed slices showed that rat Mapt protein levels were completely abolished in the Cy23 allele, suggesting a null phenotype. Finally, we intercrossed Cy23 rats to the Tg12099 rat line to generate a model of human tauopathy without endogenous rat Mapt.

### Neuropathology of Tg12099 rats

Formalin-fixed hemi-brains were coronally coarse cut and embedded into a paraffin block. Sections were cut using a microtome set at an 8-μm thickness. Brain sections were then mounted on positively charged glass slides. The sections were deparaffinized and stained with hematoxylin and eosin (H&E) or processed for IHC. For immunofluorescent procedures, after removal of paraffin with xylenes and a graded series of alcohols, heat-induced antigen retrieval was performed with citrate buffer (American MasterTech Scientific) for 20 min. Tissue sections were placed in an Autostainer 480S (Thermo Fisher Scientific) where they were incubated in 10% (v/v) normal goat serum (Vector Laboratories) made in 0.1 M phosphate-buffered saline (PBS) containing 0.2% Tween 20 (PBST) for 30 min. After washing in PBST, the sections were incubated in a primary antibody cocktail (refer to Supplementary Table S2) for 2 h. Sections were washed in PBST and incubated in species-compatible secondary antibodies (Supplementary Table S2) for 2 h at room temperature. Sections were then rinsed in PBST, added to double distilled water (ddH_2_O), subjected to a Hoechst stain (Invitrogen) for 10 min, and coverslips were mounted with PermaFluor mounting medium (Thermo Fisher Scientific). For imaging, we used the Zeiss Axio Scan.Z1 slide scanner to perform bright-field or fluorescence microscopy of the entire tissue slice using a dry 20× objective. Images were processed and analyzed with Zeiss ZEN Blue software with the analysis module.

### Thioflavin S and PBB3 staining

For maximizing tissue adherence, sections were baked at 60 °C for 15 min followed by incubation in xylene and a graded series of ethanol. Slides were then rinsed under distilled water (dH_2_O) for 2 min and placed in a solution of 1.5 μM thioflavin S (ThioS) and 50% ethanol for 10 min. Slides were rinsed in dH_2_O and then in ddH_2_O. Coverslips were mounted onto slides with PermaFluor mounting medium (Thermo Fisher Scientific). For staining with PBB3, formalin-fixed paraffin-embedded rat brain slices were photobleached overnight using a multispectral LED lamp. Next, slides were deparaffinized, washed, and permeabilized with Triton X-100 for 30 min. Slides were incubated with 2.5 μM of PBB3 (custom-synthesized) in 1× PBS for 30 min then washed twice in PBS and mounted in PermaFluor mounting medium (Thermo Fisher Scientific). Confocal z-stacks of PBB3-stained deposits were acquired using a 405-nm laser excitation and a 410–490-nm emission bandwidth and a 40× 1.1 NA water-immersion objective with 1024 × 1024 pixel resolution on a Leica SP8 confocal microscope.

### In situ hybridization (BaseScope) for human *MAPT* transcripts

Formalin-fixed paraffin-embedded rat brains were sectioned coronally at 8 µm. Tissue sections were baked at 60 °C and deparaffinized through xylenes to 100% ethanol. Endogenous peroxidases were blocked using H_2_O_2_ (ACDBio #322381). Target retrieval was performed in a steamer using target retrieval reagent (ACDBio #322000), and protease digestion was performed using Protease III (ACDBio #322381). Sections were hybridized at 40 °C to BaseScope Probe BA-Hs-MAPT-E13E14 *Homo sapiens MAPT* transcript variant 1 messenger RNA (ACDBio #723071). The probe signal was amplified, and the background was suppressed using instructions for BaseScope Detection Reagents v2 RED (ACDBio #323910). Signal was detected using the Fast Red dye included in the detection kit. Slides were counterstained with Gill Hematoxylin (Epredia #72411), and coverslips were applied using VectaMount Express mounting medium (Vector Laboratories #H-5700). For imaging, we used the Zeiss Axio Scan.Z1 slide scanner to perform bright-field microscopy of the entire tissue slice using a dry 20× objective. Images were processed with Zeiss ZEN Blue software.

### Bielschowsky silver stain to detect NFTs

Sectioned tissue was deparaffinized and washed three times in dH_2_O. Slides were then prewarmed to 40 °C and incubated in a 20% (v/v) AgNO_3_ solution for 16 min until sections became light brown in color. Slides were then added to a dH_2_O container and washed three times. Concurrently, concentrated NH_4_OH (28–30%) was added drop by drop to the 20% AgNO_3_ solution until the precipitate that formed cleared.

Slides were placed back in this H_4_AgN solution and baked in a 40 °C oven for 30 min or until sections became dark brown. Following incubation, slides were placed in developer solution for 1 min or less (using microscopy to determine exact incubation time). The developer stock solution is made fresh for each experiment and contains the following: 20 mL of 37–40% formaldehyde, 0.5 g citric acid (trisodium dihydrate; CAS 6132-04-3), 2 drops of concentrated (70%) nitric acid, and 100 mL of dH_2_O. Next, slides were dipped for 1 min in a 1% NH_4_OH (v/v) solution to stop the silver reaction and were washed in three changes of dH_2_O. Slides were then placed in 5% (v/v) Na_2_S_2_O_3_ solution for 5 min, washed in three changes of dH_2_O, put through a dehydration series, and mounted with resinous medium. For imaging, we used the Zeiss Axio Scan.Z1 slide scanner to perform bright-field microscopy of the entire tissue slice using a dry 20 × objective. Images were processed with Zeiss ZEN Blue software.

### Tissue homogenization

Whole brains or brain regions were weighed and added to a sterile 2-mL microfuge tube containing stainless steel ball bearings 2.3 mm in diameter (BioSpec Products) and ice-cold PBS with proteinase and phosphatase inhibitors (Thermo Fisher Scientific) to produce a 10% (w/v) brain homogenate. Tissue was homogenized with a Precellys 24 instrument (Bertin Technologies) set at 6,200 rpm for 80 s per round. This was repeated a total of four times with a 5-min incubation step on ice before each round of homogenization. The 10% brain homogenate was diluted 1:10 in PBS and quantified with a bicinchoninic acid (BCA) assay kit (Thermo Fisher Scientific). For the study of brain quadrants, a razor blade was used to halve the brain hemispheres and then separate the forebrain from the hindbrain. The procedures for homogenization and quantification of brain quadrants used are outlined above.

### Immunoblotting

To determine approximate levels of tau overexpression in the brains of Tg12099 rats, various concentrations of total protein from brain homogenates were added to 1× LDS sample buffer (Thermo Fisher Scientific), boiled, and applied to a 4–12% Bis–Tris PAGE gel (Thermo Fisher Scientific). Resolved proteins on gels were transferred to polyvinylidene fluoride and probed with the Tau5 primary antibody using standard Western blotting procedures. Densitometry was determined using ImageJ (v.2.0.0). To determine the approximate level of overexpression, we calculated the concentration of brain homogenate for both Tg12099(+/+) and Tg12099(+/−) rats that produced a signal equivalent to 20 μg of brain homogenate from a WT Sprague–Dawley rat. For all other blots, tissue was homogenized, and a 40-µg sample was loaded on the gels. For a complete list of primary and secondary antibodies, refer to Supplementary Table S2.

### Sarkosyl-detergent extraction of insoluble hTau species

To isolate insoluble hTau from the brains of Tg12099 rats, 200 μL of 10% brain homogenate was combined with 200 μL of extraction buffer (50 mM Tris, pH 8; 10 mM NaCl; 2 mM MgCl_2_; 4% sarkosyl; 20% sucrose; proteinase and phosphatase inhibitors; and 7.5 U/mL benzonase) and shaken at 37 °C for 30 min. Samples were then centrifuged at 20,000×*g* at 4 °C for 15 min to isolate the soluble pellet (P1). Supernatant was collected and added to a new tube and centrifuged at 100,000×*g* at 4 °C for 1 h to collect the insoluble pellet (P2). The P1 pellet was resuspended in 100 μL of PBS. The P2 pellet was washed with 200 μL of PBS and centrifuged at 100,000×*g* for 1 h at 4 °C. Supernatant was removed, and the P2 pellet was resuspended in 100 μL of PBS. P1 or P2 (5 μL) was combined with 1× LDS sample buffer and applied to PAGE gels for immunoblotting.

### Generation of recombinant tau fibrils and in vitro fibrillization

The expression and purification of recombinant K18-P301L protein were performed as described previously [36]. Briefly, *Escherichia coli* BL21-CodonPlus (DE3)-RP competent cells (Agilent) were transformed with a pRK172 plasmid encoding the tau repeat domain, K18 (residues 244–372), containing the P301L mutation. Terrific broth cultures (1 L) supplemented with 100 mg/L ampicillin and 50 mg/L chloramphenicol were inoculated with 20 mL of starter cultures and grown for 8 h. The cultures were induced with 1 mM isopropyl β-D-1-thiogalactopyranoside and grown for another 16 h. The cells were then harvested by centrifugation at 5,000×*g*, resuspended in 1 mM MES (pH 6.8), 1 mM EGTA, 0.2 mM MgCl_2_, 5 mM dithiothreitol (DTT), and 1 × cOmplete protease inhibitor cocktail (Roche), and lysed by sonication. Following the addition of 0.5 M NaCl, the lysates were boiled for 20 min and centrifuged at 48,400×*g*. The cleared lysates were dialyzed against SPFF A (20 mM MES, 50 mM NaCl, 1 mM EGTA, 1 mM MgCl_2_, 2 mM DTT, 0.1 mM phenylmethylsulfonyl fluoride, pH 6.8) and applied to a cation exchange column (SP Sepharose Fast Flow; GE Healthcare). Fractions were eluted by a NaCl gradient, and those containing K18 were concentrated (Vivaspin; GE Healthcare) and further purified by size-exclusion chromatography in PBS with 1 mM DTT (HiLoad 26/600 Superdex 200 pg; GE Healthcare). Peak fractions were analyzed by SDS-PAGE, and fractions containing > 95% tau K18 were pooled, snap-frozen, and stored at − 80 °C.

K18-P301L fibrils were generated with heparin as previously described [34, 59, 63].

### Preparation of brain-derived inoculum and intracerebral inoculations

Frozen human and rat brain tissues were thawed and weighed to determine the mass in grams. Tissue was mechanically homogenized in calcium- and magnesium-free PBS to 10% (w/v) and then subjected to limited proteinase K (PK) digestion and phosphotungstic acid (PTA) precipitation to enhance infectivity [6, 20]. Briefly, sarkosyl and benzonase were added to 10% (w/v) homogenates to final concentrations of 2% (w/v) and 150 U/mL, respectively, and incubated at 37 °C for 2 h with constant shaking at 850 rpm. PK was then added to a final concentration of 20 μg/mL, and samples were incubated for 1 h at 37 °C. To halt digestion, phenylmethylsulfonyl fluoride was added to a final concentration of 1 mM. PTA at pH 7.0 was then added to the samples to a final concentration of 2% (v/v), and the samples were incubated overnight at 37 °C with shaking at 850 rpm. Samples were then centrifuged for 30 min at 16,000×*g*, and the pellets were resuspended in PBS to 10% of the starting volume of brain homogenate. At approximately 2 months, rats were anesthetized with isoflurane and bilaterally inoculated in the thalamus with 20 μL of sample at each injection site (40 μL total per rat). Rats were assessed twice a week for signs of neurological illness, based on standard diagnostic criteria for prion disease [15]. Rats were euthanized using CO_2_ gas followed by a bilateral thoracotomy at various time points, and brains were split into quadrants as described above. The left hemisphere was frozen for biochemical analysis, and the right hemisphere was drop-fixed in formalin for 24 h and then stored in PBS for neuropathology. For the number of rats collected at each time point, refer to Supplementary Table S3.

### Human embryonic kidney (HEK293T) cell culture

Lipofectamine 2000 (Thermo Fisher Scientific) was used to transfect HEK293T cells (ATCC) with a construct that expressed hTau 0N4R containing the P301S mutation with yellow fluorescent protein (YFP) fused to the C-terminus. A monoclonal cell line referred to as HEK(P301S)-YFP was established by serial dilution and maintained in culture medium containing Dulbecco’s modified Eagle medium, 10% (v/v) fetal bovine serum, and 1% (v/v) penicillin and streptomycin. This stable cell line was used to a maximal passage number (40), at which time frozen stocks kept in liquid nitrogen were thawed for a series of quality assurance assays before experimental use. To measure tau prions in Tg or WT rats, 10% (w/v) brain homogenate was clarified by centrifugation at 1000×*g* for 5 min to remove cellular debris and large chunks of unhomogenized tissue and normalized for protein concentration using a commercially available BCA assay kit (Thermo Fisher Scientific). Transfection protein complexes containing a final concentration of 1.25 µg/mL brain homogenate lysate, Lipofectamine 2000, and Opti-MEM were incubated in 384-well plates containing HEK(tau 0N4R*P301S)-YFP or HEK(tau-4R repeat domain*P301L/V337M)-YFP cells with a cell density of 5000 cells/well for 3 days at 37 °C. Using an IN Cell Analyzer 6000 Cell Imaging System (GE Healthcare), tau intracellular aggregates were imaged and quantified with accompanying GE Healthcare software and algorithms.

### Human tissue samples

All tissue donors provided written or verbal consent to donate autopsied brains for use in biomedical research in accordance with the standards of each institution. Fresh-frozen autopsied brain tissues from neuropathologically confirmed cases of AD or PSP were procured from the Neurodegenerative Disease Brain Bank at the University of California, San Francisco or from the brain bank at Uppsala University (Sweden).

### Statistical analysis

Statistical analyses were performed using GraphPad Prism version 10 (GraphPad Software). Data are shown as mean ± standard error of mean. Comparisons between two groups were performed by unpaired *t* test with Welch correction. Comparisons between multiple groups were performed by mixed-model analysis with the post-hoc Dunnett’s test or the Tukey’s multiple comparison test. For Kaplan–Meier survival analysis, we used the log-rank Mantel–Cox test to compare groups. Sample sizes are reported in the figure legends.

## Results

### Establishing 0N4R MAPT*P301S rats to model human tauopathies

**Fig. 1:**
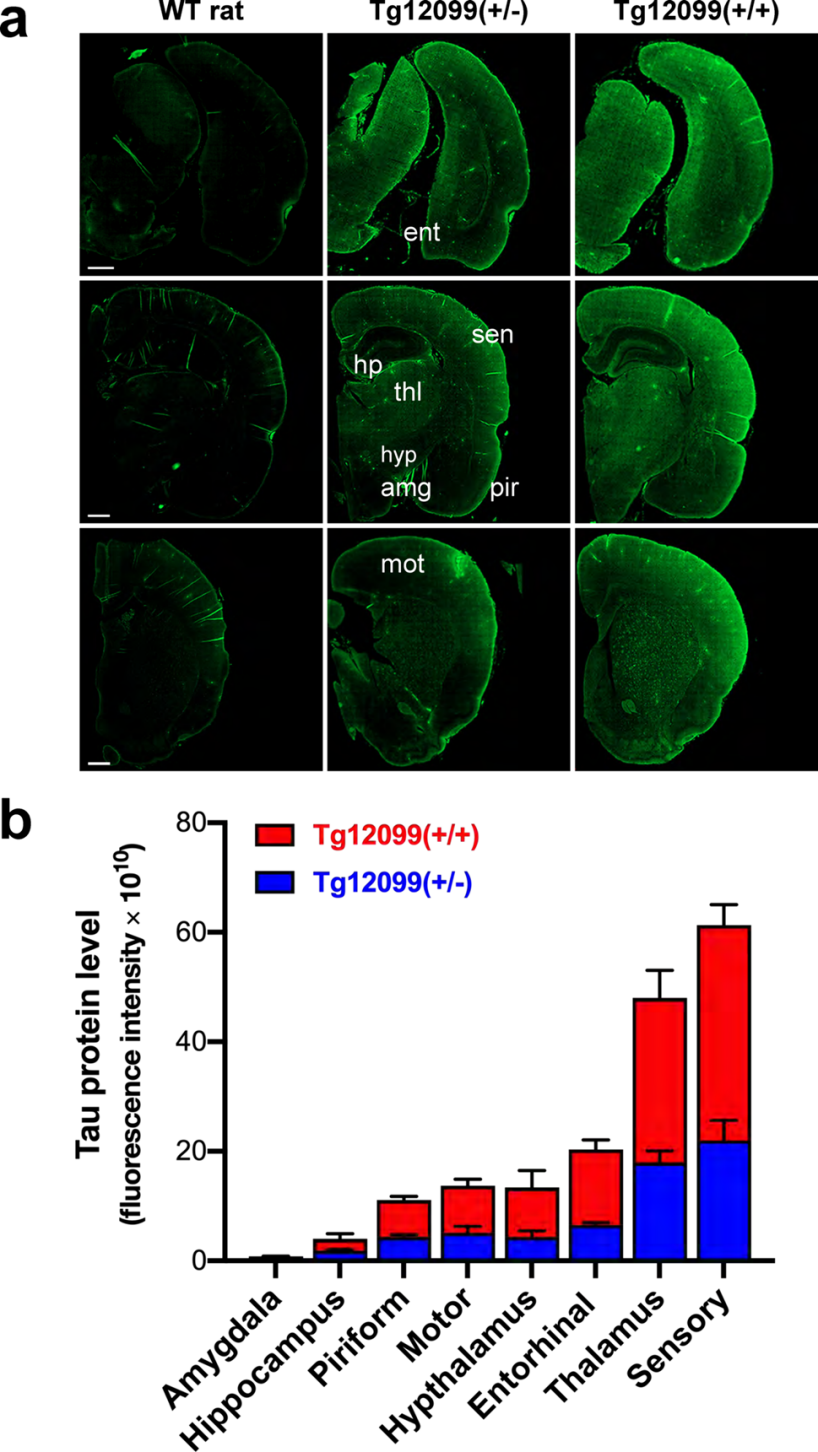
MAPT*P301S protein expression in the brains of Tg12099 rats. **a** Coronal rat brain sections stained with Tau13 (total human tau, hTau) to detect MAPT*P301S protein expression via immunofluorescence. Left images: 3-month-old wild-type (WT) rat; middle images: age-matched Tg12099(+/−) rat; right images: Tg12099(+/+) rat. Top images are at the entorhinal cortex level, middle images are at the hippocampus level, and the bottom images are at the striatum level. While nonspecific fluorescence of hTau was detected in WT rats, Tg12099(+/−) rats had hTau expression in multiple brain regions, which was increased in Tg12099(+/+) rats. Brain regions: amg, amygdala; ent, entorhinal cortex; hp, hippocampus; hyp, hypothalamus; mot, motor cortex; pir, piriform cortex; sen, sensory cortex; thl, thalamus. All scale bars = 1000 μm. **b** Quantification of hTau expression represented as summed fluorescence intensity by brain region demonstrates higher MAPT*P301S protein expression in most brain regions of Tg12099(+/+) rats (red bars) compared with Tg12099(+/−) rats (blue bars). For background correction, the mean fluorescence intensity of WT rats (*n* = 3) was subtracted from the summed fluorescence values of Tg12099(+/−) and Tg12099(+/+) rats (*n* = 3 rats per genotype). Data represent mean ± SEM of summed fluorescence intensities

**Fig. 2:**
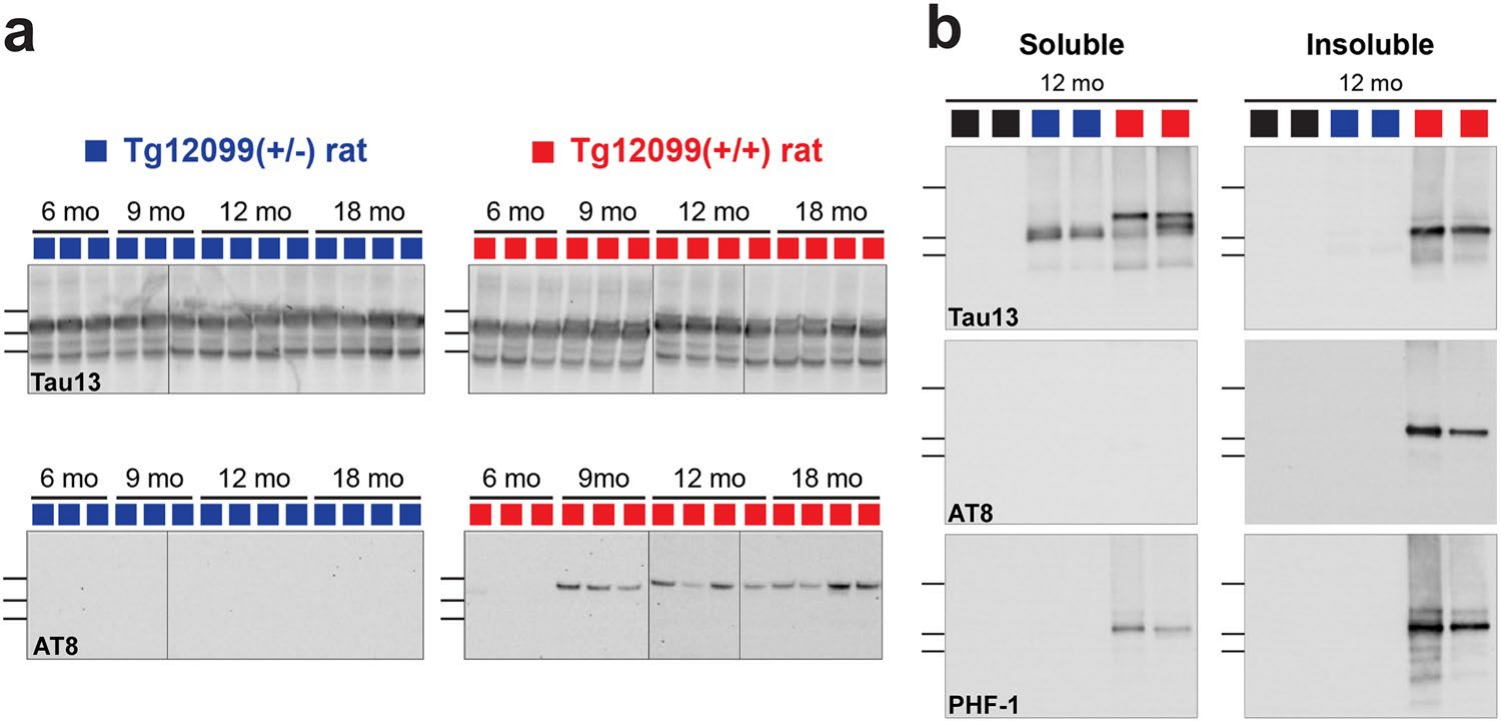
Hyperphosphorylation of MAPT*P301S coincides with the formation of insoluble hTau species in Tg12099(+/+) rats. **a** Time course comparing total hTau to AT8. Brain homogenates derived from Tg12099(+/−) or Tg12099(+/+) rats were immunoblotted with Tau13 to detect hTau (top blots) or AT8 (bottom blots). Markers from top to bottom are 60 kDa, 50 kDa, and 40 kDa. **b** Sarkosyl extraction of soluble or insoluble hTau protein from 12-month-old Tg12099 or WT rat brain homogenates immunoblotted with Tau13, AT8, or PHF-1 antibodies. Blue square: one Tg12099(+/−) rat brain sample; red square: one Tg12099(+/+) rat brain sample; black square: one WT rat brain sample. *mo* month

**Fig. 3:**
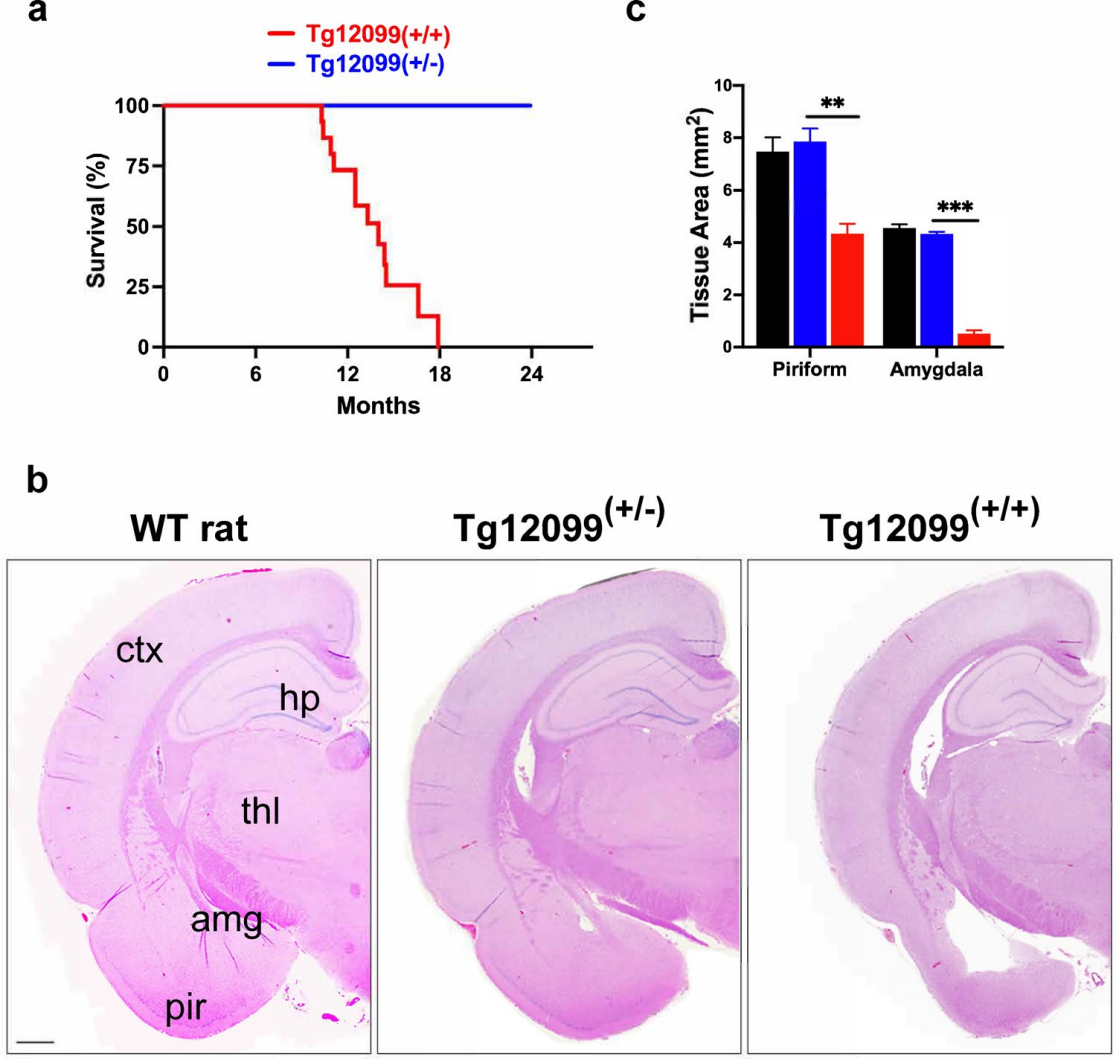
Focal neurodegeneration in aged Tg12099(+/+) rats. **a** Kaplan–Meier plot depicts survival percentage of Tg12099(+/+) rats (*n* = 13) compared with Tg12099(+/−) rats (*n* = 16). **b** Hematoxylin and eosin staining was performed on terminal Tg12099(+/+) rats (*n* = 8), age-matched Tg12099(+/−) rats (*n* = 4), and age-matched WT rats (*n* = 3) to detect neurodegeneration. Coronal section of a 16-month-old Tg12099(+/+) rat brain (right image) shows significant deterioration of the amygdala and piriform cortex in addition to hydrocephaly. Coronal section of an 18-month-old WT rat (left image) or an age-matched Tg12099(+/−) rat (middle image) did not display these neurological features. Amg, amygdala; ctx, cortex; hp, hippocampus; pir, piriform cortex; thl, thalamus. Scale bar = 1000 μm. **c** Quantification of tissue surface area shows a reduction in the piriform cortex and amygdala in terminal Tg12099(+/+) rats (red bars) compared with age-matched Tg12099(+/−) and WT rats (blue and black bars, respectively). Data represent mean ± SEM (*n* = 4–6 rats per genotype; **P < 0.005 and ***P < 0.0001). Each rat genotype was compared with a corresponding brain region followed by an unpaired *t* test analysis with Welch correction

To establish a tauopathy model in rats, we created a construct that uses the rat PrP promoter, *RaPrnp* [56], to overexpress the human 0N4R tau isoform with the MAPT*P301S mutation in the CNS of rats. The resulting RaPrnp-MAPT*P301S construct was microinjected into Sprague–Dawley rat embryos to generate the Tg12099 line (Supplementary Fig. S1a), which harbors 21 to 22 copies of the transgene as measured by ddPCR (Supplementary Fig. S1a). Because transgene insertion into the genome is random, there is a possibility that insertion can occur in a genetic locus that will influence phenotypic analysis. We used TLA to map transgene insertion sites and define the RaPrnp-MAPT*P301S insertion site in Tg12099 rats [14, 52]. TLA and next-generation sequencing revealed a single insertion site of RaPrnp-MAPT*P301S into the rat chromosome 18:20,312,421. There was no evidence of insertion into a protein-coding gene sequence or local genomic structural rearrangements (Supplementary Fig. S1b, c). Importantly, NM_001287621 and *Pik3c3* were distant to the transgene insertion site at 1 and 2.6 megabases, respectively (Supplementary Fig. S1c). To determine the regional expression of the MAPT*P301S transgene in Tg12099(+/−) and Tg12099(+/+) rats, we immunostained brain sections with the Tau13 monoclonal antibody, which targets human-specific total tau. This revealed ubiquitous hTau protein expression in the adult CNS compared with age-matched WT rats (Fig. 1a, b). By quantifying the fluorescence intensity of Tau13 staining, we determined that Tg12099(+/+) rats had higher levels of MAPT*P301S expression in the hippocampus and hypothalamus as well as the thalamus and entorhinal, motor, piriform, and sensory cortices compared with Tg12099(+/−) animals (Fig. 1b). The amygdala in either genotype had the weakest expression of MAPT*P301S (Fig. 1b). Immunoblotting with Tau13 also demonstrated stable hTau expression up to 18 months of age in Tg12099(+/−) and Tg12099(+/+) rats (Fig. 2a, top images). In both genotypes, the hTau protein migrated as a predominant band that was slightly above the ~ 50 kDa marker and as a band below the ~ 40 kDa marker (Fig. 2a, b). To semiquantitatively assess the overexpression of hTau in the brains of Tg12099 rats, we immunoblotted using the Tau5 antibody that detects both human and rat tau. We found that tau expression was approximately 1.5-fold and 4.5-fold higher in the brains of Tg12099(+/−) and Tg12099(+/+) rats, respectively, than endogenous tau expression in the brains of WT Sprague–Dawley rats (Supplementary Fig. S2). Next, we performed in situ hybridization (BaseScope) using a probe specific for human *MAPT* to visualize single RNA transcripts at the cellular level. We confirmed the gene-dose dependence of expression in the piriform cortices of advanced-age rats. Moreover, we also observed that most of the RNA transcripts appeared to localize to cells with neuronal morphology based on the hematoxylin counterstain (Supplementary Fig. S3). While Tg12099(+/−) rats could live over 24 months, the median survival of Tg12099(+/+) rats was 14 months (Fig. 3a). Aging Tg12099(+/+) rats exhibited progressive neurological deficits including ataxia (*n* = 3), bradykinesia (*n* = 3), and seizures (*n* = 8). Rats displaying two or more of these clinical signs and showing no signs of improvement within 24 h were euthanized to examine biochemical and neuropathological changes of hTau.

### Focal neurodegeneration of the corticolimbic system in terminal Tg12099(+/+) rats

To determine if the 0N4R MAPT*P301S transgene was responsible for the observed neuroclinical phenotypes, we conducted neuropathology with H&E staining in ill Tg12099(+/+) rats and age-matched WT and Tg12099(+/−) rats. Terminal Tg12099(+/+) rat brains displayed focal neurodegeneration in the forebrain, predominantly in the piriform cortex with a near loss of the amygdala and an enlarged ventricular space, which was not seen in WT and Tg12099(+/−) rats (Fig. 3b and Supplementary Fig. S4a). Importantly, no significant differences in brain area were found between WT and Tg12099(+/−) rats (Fig. 3b, c). By quantifying tissue area in Tg12099(+/+) rats, we observed a reduction of approximately 46% in the piriform cortex with greater atrophy in the amygdala (~ 88%) compared with Tg12099(+/−) rats (Fig. 3c). Less affected regions showed focal vacuolization throughout the cortices of Tg12099(+/+) rats compared with Tg12099(+/−) rats (Supplementary Fig. S4b). Sporadic vacuolization was also observed in the brainstems of Tg12099(+/+) rats with only a few vacuoles apparent in Tg12099(+/−) rats (Supplementary Fig. S4b). Thus, MAPT*P301S overexpression caused neurodegeneration and lethality in Tg12099(+/+) rats.

### Neuronal loss and gliosis in the cortical and limbic systems of terminal Tg12099(+/+) rats

**Fig. 4:**
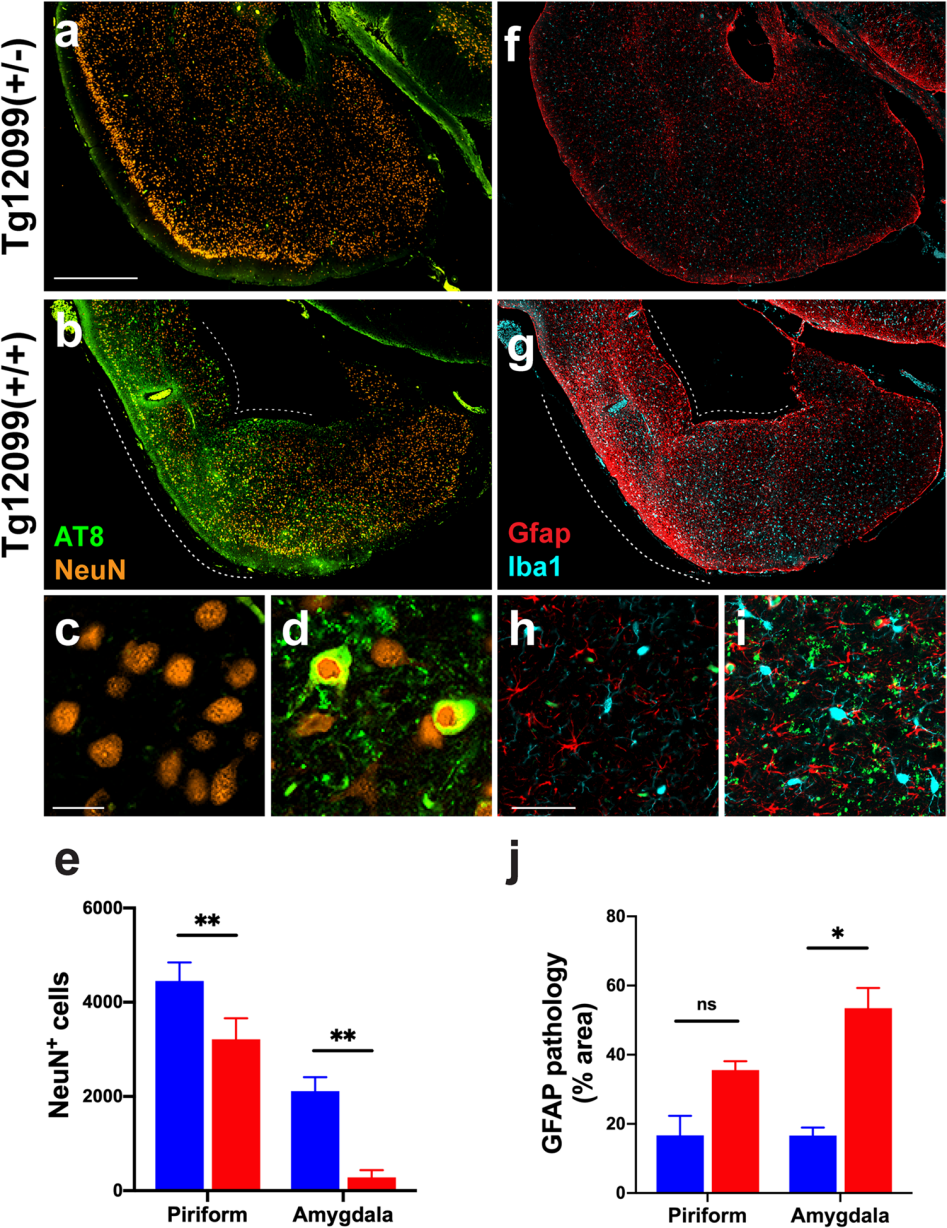
Neuronal loss and gliosis in terminal Tg12099(+/+) rats. **a**–**d** Piriform cortex and amygdala stained for AT8 (green) and NeuN (orange). **a**, **b** Low magnification of a terminal Tg12099(+/+) rat in (**b**) exhibits a dense AT8^+^ region (dotted line) compared with a Tg12099(+/−) rat in (**a**). Scale bar = 1000 μm. **c**, **d** Higher magnification of the piriform cortex in (**b**) shows AT8^+^ inclusions in neurons and surrounding neuropil (**d**) compared with the Tg12099(+/−) rat in (**c**). Scale bar = 10 μm. **e** Quantification of NeuN^+^ cells in the piriform cortex and amygdala in terminal Tg12099(+/+) rats (red) compared with age-matched Tg12099(+/−) rats (blue). **f**–**i** Piriform cortex and amygdala stained with Gfap (red) for astrocytes and Iba1 (turquoise) for microglia. **f**, **g** Low magnification of a terminal Tg12099(+/+) rat in (**g**) compared with the Tg12099(+/−) rat in (**f**). Scale bar = 1000 μm. **h**, **i** Higher magnification of the piriform cortex in (**g**) shows that AT8^+^ inclusions colocalize with activated astrocytes and reactive microglia (**i**) compared with the Tg12099(+/−) rat in (**h**). Scale bar = 25 μm. **j** Quantification of Gfap^+^ percent area in Tg12099(+/+) rats (red) compared with Tg12099(+/−) rats (blue). Data represent mean ± SEM (*n* = 4–6 rats per genotype; ns = not significant, *P < 0.05, and **P < 0.001). Each rat genotype was compared with a corresponding brain region followed by an unpaired *t* test analysis with Welch correction

Because abnormally phosphorylated tau (pTau) is a hallmark of AD and primary tauopathies [7, 37, 45, 68], we investigated whether the transgene product, MAPT*P301S protein, undergoes post-translational modification in the Tg12099(+/+) rats. We used an AT8 antibody to detect pTau (S202 and T205) and NeuN to detect neurons. We observed reductions of approximately 28% and approximately 87% of NeuN^+^ neurons in the piriform cortex and amygdala, respectively, in terminal Tg12099(+/+) rats compared with age-matched 18-month-old Tg12099(+/−) rats (Fig. 4a–e). Gliosis has been observed to accompany neuronal loss in the tauopathies, so we investigated whether this was also true in our rat model. We observed an increasing trend of the percent area of reactive astrocytes stained for Gfap in the piriform cortex and amygdala of Tg12099(+/+) rats compared with Tg12099(+/−) rats (Fig. 4f–j). These neuropathological observations reveal that these highly specific brain regions in Tg12099(+/+) rats are vulnerable to the human MAPT*P301S mutation.

### Tau hyperphosphorylation is restricted to the forebrain and limbic system in Tg12099(+/+) rats

**Fig. 5:**
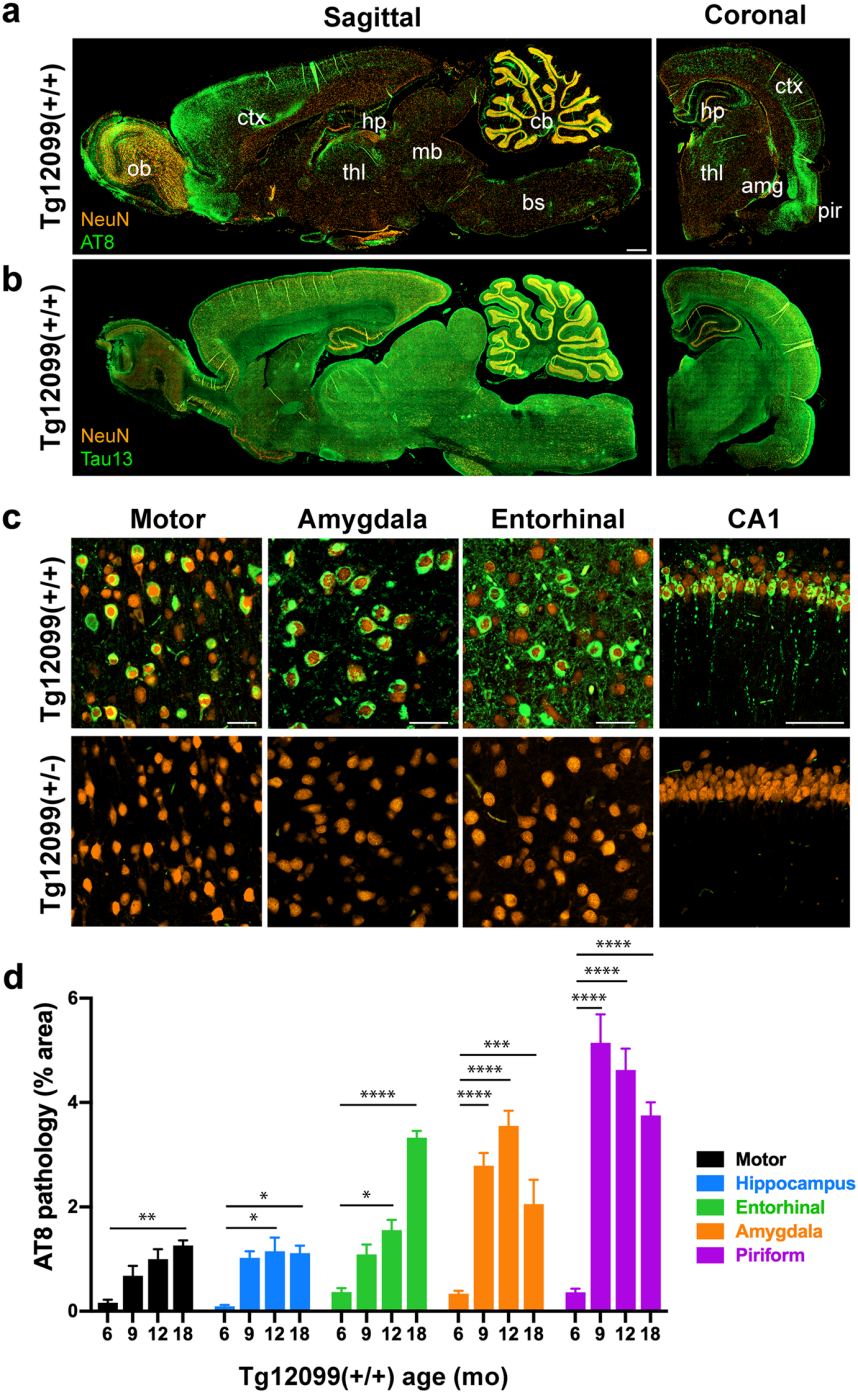
Tg12099(+/+) rats overexpressing a familial MAPT*P301S mutation have progressive hTau hyperphosphorylation in the forebrain and limbic system. **a** Sagittal (left image) and coronal (right image) sections of a 12-month-old Tg12099(+/+) rat brain display regional AT8^+^ (green) accumulation in the frontal cortex and in the limbic, midbrain, and thalamic structures. The nuclei of neurons are stained for NeuN (orange). Left side of sagittal image in (**a**) is forebrain; right is hindbrain. **b** Sagittal (left image) and coronal (right image) sections of the same rat were stained for NeuN and Tau13 to detect total hTau protein expression. Amg, amygdala; bs, brainstem; cb, cerebellum; ctx, cortex; hp, hippocampus; mb, midbrain; ob, olfactory bulb; pir, piriform cortex; thl, thalamus. Scale bar = 1000 μm in (**a**) also corresponds to (**b**). **c** Higher magnification of AT8^+^ and NeuN staining. A Tg12099(+/+) rat (top panels) compared with a 12-month-old Tg12099(+/−) age-matched rat (bottom panels). Scale bar = 50 μm in images from the motor cortex, amygdala, and entorhinal cortex. Scale bar = 100 μm in image from CA1. **d** Quantification of AT8^+^ pathology as percent area in the corticolimbic system of Tg12099(+/+) rats (*n* = 3–6 rats per age in months [mo]; mean ± SEM; *P < 0.05, **P < 0.01, ***P < 0.001, and ****P < 0.0001). Each time point per brain region was compared using a mixed-effects analysis followed by Dunnett's multiple comparisons test

Since we observed abundant AT8^+^ neurons in the atrophied piriform cortex and amygdala of terminal Tg12099(+/+) rats (Fig. 4b, c), we sought to determine the temporal progression and spatial distribution of this key tauopathy hallmark. First, we studied the regional distribution of tau tangles in both sagittal and coronal brain slices from late-stage Tg12099(+/+) rats. We observed dense AT8^+^ deposition in the forebrain and limbic structures (Fig. 5a) despite high levels of hTau protein expression (Tau13 staining) in multiple brain regions (Fig. 5b). Curiously, we observed little AT8^+^ staining in hindbrain structures, including the posterior midbrain, cerebellum, and brainstem (Fig. 5a). Higher magnification of samples from terminal Tg12099(+/+) rats revealed dense AT8^+^ inclusions in cell bodies, including intense staining of the neuropil in the amygdala, cortex, and hippocampus, compared with Tg12099(+/−) rats (Fig. 5c). We used a digital slide scanner and analysis algorithm to quantify the abundance of AT8^+^ tau in brain regions functionally relevant to primary tauopathies. In Tg12099(+/+) rats, we did not observe any AT8^+^ staining at 3 months of age. However, at 6 months of age, we detected AT8^+^ neuronal inclusions in the cortex and hippocampus of Tg12099(+/+) rats (Fig. 5d). These inclusions progressed until late terminal stages through 12 and 18 months (Fig. 5d). Interestingly, the amygdala and piriform cortex had the highest abundance of AT8^+^ inclusions at 12 and 9 months, respectively. However, we found a significant reduction of AT8^+^ staining in these regions by 18 months of age (Fig. 5d), which may be due to advanced neurodegeneration and clearance of ghost tangles (Fig. 3b and Supplementary Fig. S4a). In contrast to the Tg12099(+/+) rats, the Tg12099(+/−) rats exhibited only a few AT8^+^ neurons in the entorhinal cortex at 18 months of age (Fig. 4a).

The appearance of an AT8 band migrating between the 50 kDa and 60 kDa markers was first detected in the brains of 9-month-old Tg12099(+/+) rats following immunoblotting and continued to be present at 12–18 months (Fig. 2a, lower images), confirming our neuropathological profiling. Despite hTau being expressed throughout the lifetime of Tg12099(+/−) rats, no discernable AT8 band was observed up to 18 months in these rats (Fig. 2a, lower images). Because hyperphosphorylation of tau is associated with sarkosyl insolubility [57, 61], we examined this phenotype in Tg12099 rats using sarkosyl detergent extraction. Using the Tau13 antibody, we detected hTau migrating at 50–55 kDa in the soluble fractions of 12-month-old Tg12099(+/−) and Tg12099(+/+) rat brains (Fig. 2b, top images). Sarkosyl-insoluble hTau was only detected in Tg12099(+/+) rat brains (Fig. 2b, top images), not in Tg12099(+/−) rat brains. Because hTau is abnormally hyperphosphorylated at multiple epitopes in disease [11, 33, 69], we probed the blots with AT8 and PHF-1 (S396 and S404) and observed hyperphosphorylated hTau bands in the sarkosyl-insoluble fraction with both antibodies (Fig. 2b, middle and bottom images). Of note, a PHF-1 band was detected in the soluble fraction of Tg12099(+/+) rat brains, suggesting soluble and sarkosyl-insoluble pools of hTau are hyperphosphorylated (S396 and S404 epitopes) in diseased brains (Fig. 2b, bottom images). There were no signals detected in WT rat samples for any of the antibodies or conditions we tested (Fig. 2b).

### MAPT*P301S changes conformation to form NFTs in the frontal cortex and limbic system in Tg12099(+/+) rats

**Fig. 6:**
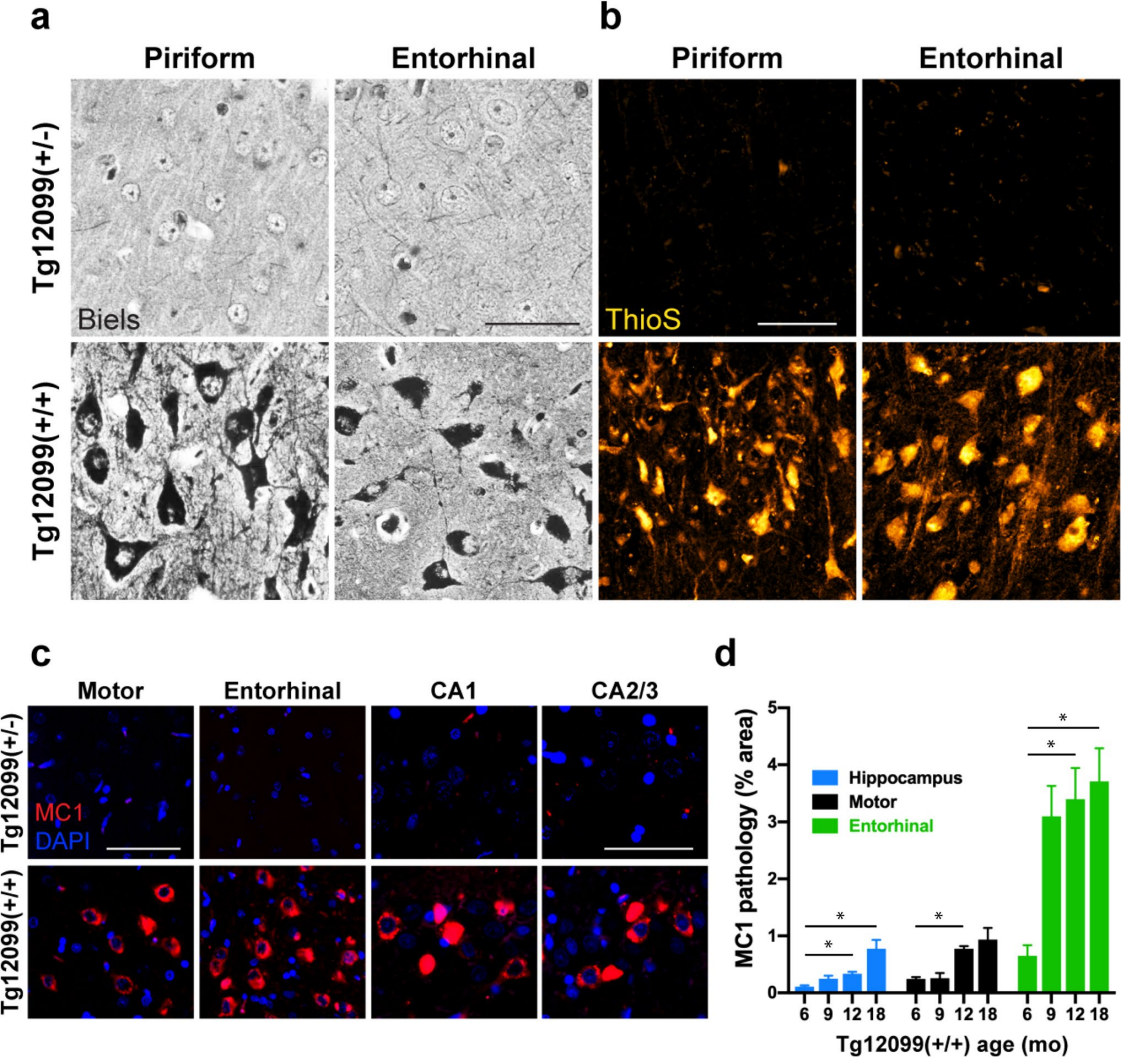
Conformational changes in hTau and the formation of neurofibrillary tangles (NFTs) in Tg12099(+/+) rats. **a** A Tg12099(+/−) rat (top panels) compared with a Tg12099(+/+) rat (bottom panels) with silver-positive NFTs in the piriform and entorhinal cortices. Scale bar = 75 μm. **b** A Tg12099(+/−) rat (top panels) compared with a Tg12099(+/+) rat (bottom panels) with thioflavin S^+^ (ThioS^+^) neurons. Scale bar = 50 μm. **c** A Tg12099(+/−) rat (top panels) compared with a Tg12099(+/+) rat (bottom panels) with MC1^+^ inclusions (red). Nuclei are counterstained with DAPI (blue). Scale bar = 50 μm in images from the motor and entorhinal cortices; scale bar = 75 μm in CA layers. **d** Quantification of MC1^+^ pathology as percent area in the hippocampus and motor and entorhinal cortices of Tg12099(+/+) rats (*n* = 3–6 rats per age in months [mo]; mean ± SEM; *P < 0.05). Each time point per brain region was compared using a mixed-effects analysis followed by Dunnett's multiple comparisons test

In AD and the primary tauopathies, the formation of NFTs is a key indicator of disease. Bielschowsky silver staining revealed dense silver-positive NFTs in the piriform and entorhinal cortices of Tg12099(+/+) rats, strikingly similar to human NFTs (Fig. 6a; compare top panels with bottom). NFTs in the piriform and entorhinal cortices also stained positive for ThioS, demonstrating the formation of β-pleated sheet structures in terminal Tg12099(+/+) rats (Fig. 6b; compare top panels with bottom). To investigate whether the tertiary structure of hTau was altered, we stained brain sections with a conformational-dependent antibody, MC1, that recognizes a discontinuous epitope consisting of the N-terminus (aa 7–9) and the three-repeat domains (aa 313–322) of hTau [46]. We observed the progressive formation of MC1^+^ neurons in the forebrain cortex and in the CA1 or CA2/3 layers of the hippocampus of Tg12099(+/+) rats but did not detect MC1^+^ neurons in 18-month-old Tg12099(+/−) rats (Fig. 6c; compare top panels with bottom). MC1^+^ aggregates, appearing as speckled inclusions or large globose-like structures, were found primarily in the cell bodies of neurons (Fig. 6c). We quantified MC1^+^ as percent area in the motor and entorhinal cortices and the hippocampus and found hTau conformational changes beginning at 6 months, which progressed until terminal stages (Fig. 6d).

To further characterize the conformational changes in tau, we assessed the labeling efficiency of the clinical positron emission tomography (PET) ligand PBB3, which has been used to visualize tau burden in human tauopathy patient samples and Tg mouse models [58, 62]. Taking advantage of the intrinsic fluorescence of the PBB3 compound, we stained fixed brain slices and performed confocal microscopy. We observed intense PBB3 colabeling of NFTs immunolabeled with AT8 pTau antibody in the frontal cortex and the hippocampus of terminal Tg12099(+/+) rats (Supplementary Fig. S5a, b).

### Deletion of endogenous rat *Mapt* does not alter the neuropathological phenotype of Tg12099 rats

In mouse models of human tauopathy, the endogenous WT murine tau is templated by and incorporates into human mutant tau deposits [39, 43, 60, 82]. This observation suggests that murine tau contributes to the pathogenic phenotype and that removing endogenous tau would aid in determining the cellular and functional consequences of the expressed human mutant tau. Indeed, several groups created human tauopathy mouse models lacking endogenous tau and found altered abundance of tau pathology [3, 47] and neurotoxicity [76] and a change in mouse survival [3]. Similarly, we established the Tg12099 line on a rat *Mapt*–null background to determine if endogenous rat tau was contributing to the tau neuropathological phenotype of our model. Because rats lacking endogenous *Mapt* were unavailable from commercial or academic sources, we custom-made our own using TALENs (see Methods and Supplementary Fig. S6a) on the Sprague‒Dawley genetic background. We confirmed gene knockout using Sanger sequencing and PAGE genotyping (Supplementary Fig. S6b, c). We bred the “Cy23” line to homozygosity and measured Mapt protein levels in brain homogenates and fixed brain slices using an antibody specific to rodent Mapt protein (T49). Data from Western blots of brain homogenates and IHC of fixed slices showed that rat Mapt protein levels were completely abolished in the Cy23 allele, suggesting a null phenotype (Supplementary Fig. S6d, e). Next, we backcrossed to the Tg12099(+/+) line (Supplementary Fig. S7). Consistent with murine *Mapt*–knockout mouse models, ablation of the rat tau gene did not alter the gross phenotypes in WT or Tg12099 rats. In general, the spatiotemporal patterns of tau pathology and associated gliosis in the Tg12099xCy23(+/+) line appeared similar to the parent Tg12099 line (Supplementary Fig. S7a–c). The pathology had the same focal predominance in frontotemporal brain regions with consequent brain atrophy in late-stage tissues (Supplementary Fig. S7c). Moreover, the formation of mature tau tangles was confirmed by ThioS (not shown) and silver staining (Supplementary Fig. S7d). Strikingly, we found that Tg12099xCy23(+/+) rats lived longer (median survival: 580 days) than the parent Tg12099(+/+) rats (median survival: 440 days) (Supplementary Fig. S7e). These data suggest that endogenous rat tau in Tg12099(+/+) contributes to the progression of tau pathology, consequent neurodegeneration, and neurological signs.

### Regional tau prion propagation in Tg12099(+/+) rats is highest in the corticolimbic system

**Fig. 7:**
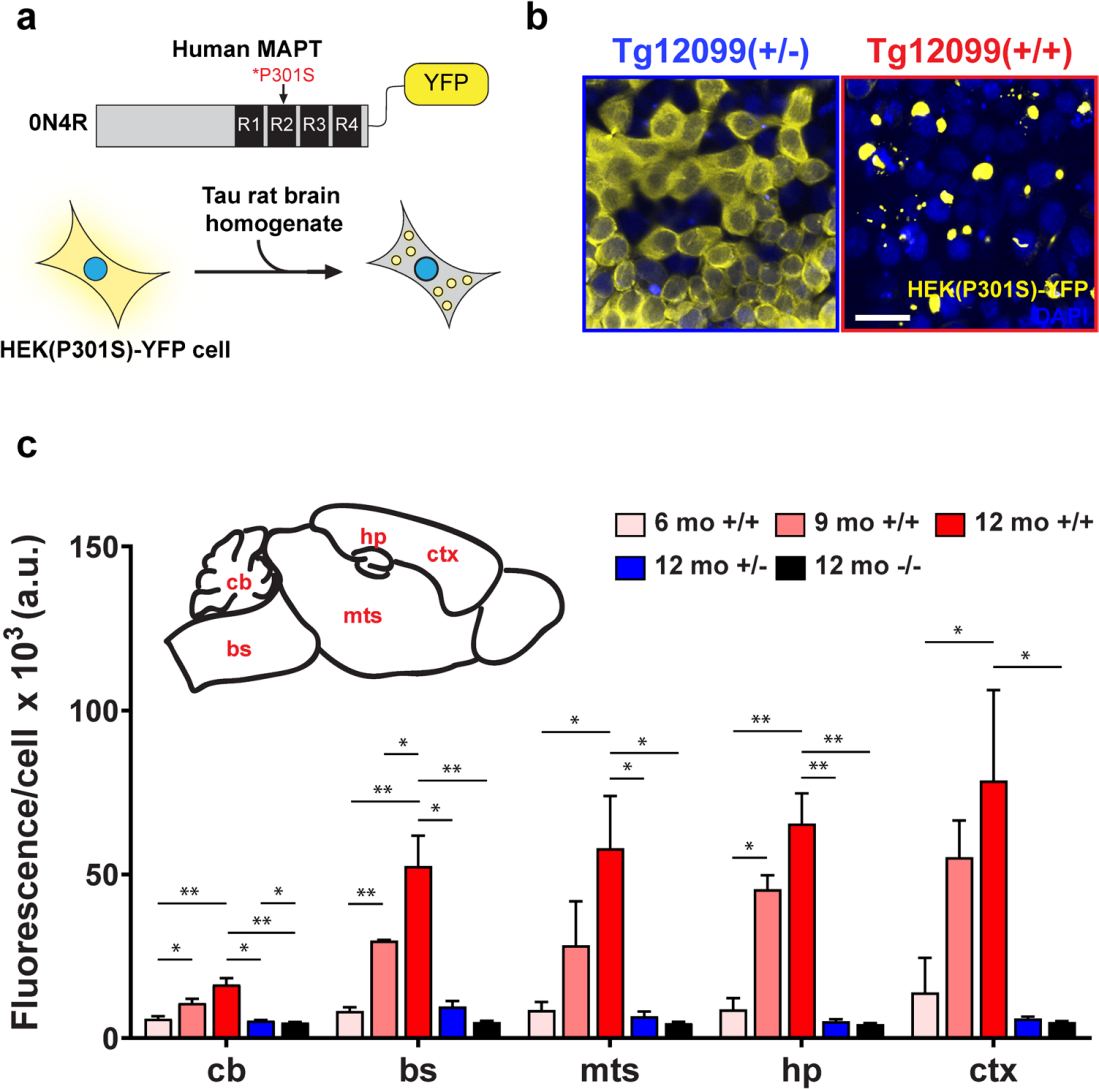
Self-propagation of tau prions in the forebrain and limbic system of Tg12099 rats. **a** Top illustration: 0N4R MAPT*P301S construct with yellow fluorescent protein (YFP) fused to the C-terminus was used to derive a monoclonal human embryonic kidney (HEK) cell line, HEK(P301S)-YFP, to quantify tau prion load in the brains of transgenic rats (*n* = 4–5 rats per time point). **b** Brain lysates from 12-month-old Tg12099(+/−) or Tg12099(+/+) rats resulted in cytoplasmic or aggregated (P301S)-YFP, respectively. The fluorescence intensity of (P301S)-YFP aggregates (yellow) per cell reflects the abundance of tau prions in an aged Tg12099(+/+) rat. Nuclei are stained with DAPI (blue). **c** Schematic depicts the five microdissected brain regions applied to the HEK(P301S)-YFP cell assay. bs, brainstem; cb, cerebellum; ctx, cortex; hp, hippocampus; mts, midbrain, thalamus, and striatum. Brain lysates from 12-month-old WT (black bars) or Tg12099(+/−) rats (blue bars) display baseline levels of tau prion cell infectivity. Tau prion load in Tg12099(+/+) rats (red bars) shows propagation in distinct brain regions over the course of 12 months. Data are represented as mean ± SEM (*n* = 3–6 rats per age in months [mo]; *P < 0.05 and **P < 0.01). Each time point per brain region was compared using a mixed-effects analysis followed by Tukey's multiple comparisons test. Further, we compared the effect of genotypes in 12-month-old rats using mixed-effects analysis followed by Tukey's multiple comparisons test. *a.u.* arbitrary unit

Given the spatiotemporal spread of misfolded hTau in the brains of Tg12099(+/+) rats, we wanted to define the regional propagation of tau prions in Tg12099 rats. We constructed a monoclonal HEK293T cell line that overexpresses hTau 0N4R isoform with the MAPT*P301S mutation and YFP fused to the C-terminus (Fig. 7a), which we refer to as HEK(P301S)-YFP cells. Importantly, the MAPT*P301S-YFP fusion protein is cytoplasmic in HEK(P301S)-YFP cells and does not spontaneously form tau prions. To globally quantify tau prion levels from whole brains, Tg12099 rat brains were collected over time, and homogenates were applied to HEK(P301S)-YFP cells. Images were captured by high-content confocal microscopy 72 h later and analyzed to measure fluorescence intensity of aggregates per cell (Fig. 7b). We observed tau prion infectivity beginning at 6 months of age, which increased linearly until terminal stages when Tg12099(+/+) rats became clinically ill (Fig. 3a and Supplementary Fig. S8). In contrast, Tg12099(+/−) rats had no detectable tau prions at any time point (Supplementary Fig. S8).

Given the global increase of tau prions in Tg12099(+/+) whole rat brains, we then wanted to identify the brain regions that were susceptible to tau prion propagation. We microdissected the brainstem, cerebellum, cortex, and hippocampus, and collected the midbrain, thalamus, and striatum as one unit (known as mts) from WT, Tg12099(+/−), and Tg12099(+/+) rats at different ages. We then exposed these tissue samples to the tau prion cell-infectivity assay. Compared with baseline infectivity in 12-month-old WT and Tg12099(+/−) rats, 6-month-old Tg12099(+/+) rats had a slight increase in tau prions in the hippocampus and cortex (Fig. 7c). Tau prion levels rapidly increased in 9- and 12-month-old Tg12099(+/+) rats—most notably in the cortex and hippocampus, followed by the mts and brainstem, with a minor increase in the cerebellum (Fig. 7c). Thus, quantifying tau prion propagation in Tg12099(+/+) rats revealed that the corticolimbic system was most vulnerable to tau prions.

### Transmission of recombinant tau prions in Tg12099(+/−) rats

**Fig. 8:**
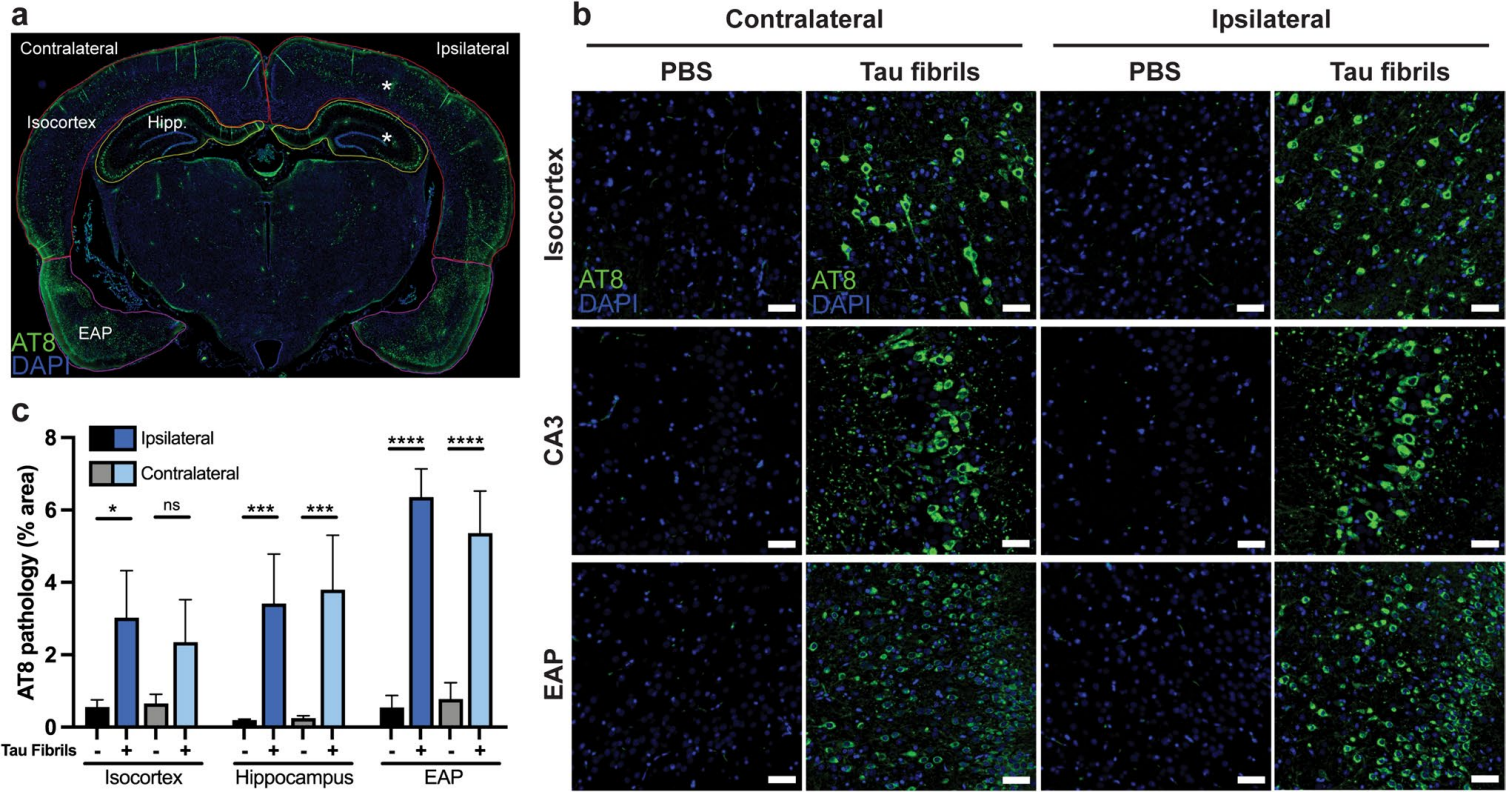
Intracerebral injection of tau fibrils into Tg12099(+/−) rats drives global hTau hyperphosphorylation at 6 months of age. **a** Diagram showing low-magnification image of a Tg12099(+/−) rat brain injected with tau fibrils at 4-months post-stereotactic surgery. Asterisks show the locations of the stereotactic injection sites in the left hippocampus and overlying cortex of Tg12099(+/−) rats at 2 months of age. Regions of interest used for quantification are demarcated in red (isocortex), yellow (hippocampus), and magenta (EAP: combination of entorhinal cortex, amygdala, and piriform cortex). Robust bilateral AT8^+^ (green) staining is observed from a unilateral injection. Nuclei (blue) are counterstained with DAPI. **b** High magnification of ipsilateral (left) and contralateral (right) images of Tg12099(+/−) rats injected with PBS or tau fibrils. AT8^+^ inclusions were detected in cell bodies and neuropil in the frontal and entorhinal cortices as well as in the CA3 layer and striatum of Tg12099(+/−) rats inoculated with tau fibrils. Aside from nonspecific staining of blood vessels, PBS-injected animals never displayed AT8^+^ inclusions. All scale bars in images = 50 μm. **c** Quantification of AT8 % area burden in the isocortex (red), hippocampus (yellow), and EAP (magenta) regions of both hemispheres ipsilateral and contralateral to the injection site. Data are represented as mean ± SEM (*n* = 3 rats per Tau K18 fibril injection group and *n* = 4 rats per PBS injection group; *P < 0.05, **P < 0.01, ***P < 0.001, and ****P < 0.0001). In each brain region, we compared the PBS-injected group versus the Tau K8 fibril–injected group using a one-way ANOVA followed by Tukey’s multiple comparisons test. There were no significant differences between ipsilateral and contralateral sides of the same region

Previous studies have demonstrated that intracerebral injection of brain homogenates from patients with human tauopathies or in vitro–synthesized tau fibrils into Tg mice can lead to the propagation and spread of tau prions [9, 17, 18, 31, 43]. To ascertain if this is a conserved mechanism among rodents, we examined whether our tauopathy rat model would be vulnerable to the inoculation of exogenous tau prions. Using Tg12099(+/−) rats, which do not display overt signs of tauopathy yet express the MAPT*P301S transgene, we unilaterally injected tau fibrils (1 mg/mL) or PBS (vehicle) into the hippocampus and overlying cortex of 2-month-old Tg12099(+/−) rats (Fig. 8a). Tg12099(+/−) rats were euthanized 4 months later to examine the regional localization of pTau via AT8 staining and neuropathology. Unlike PBS-injected control rats that lacked AT8 staining (Fig. 8b), we observed global AT8^+^ inclusions in Tg12099(+/−) rats injected with tau fibrils (Fig. 8c). Strikingly, AT8^+^ inclusions were located ipsilateral and contralateral to the injection site in the entorhinal, frontal, and piriform cortices as well as in the CA3 layer and the thalamus—regions vulnerable to spontaneous tauopathy (Figs. 4c–e and 5a–d). Under higher magnification, tau fibril–injected rats accumulated AT8^+^ inclusions in neuronal cell bodies and neuropil compared with PBS-injected controls. Since Tg12099(+/−) rats do not exhibit spontaneous tauopathy, we surmised that the expression of MAPT*P301S rendered neurons susceptible to the uptake and propagation of tau prions.

### Rapid propagation and spread of tau prions induced by recombinant tau fibrils in Tg12099(+/−) rats

**Fig. 9:**
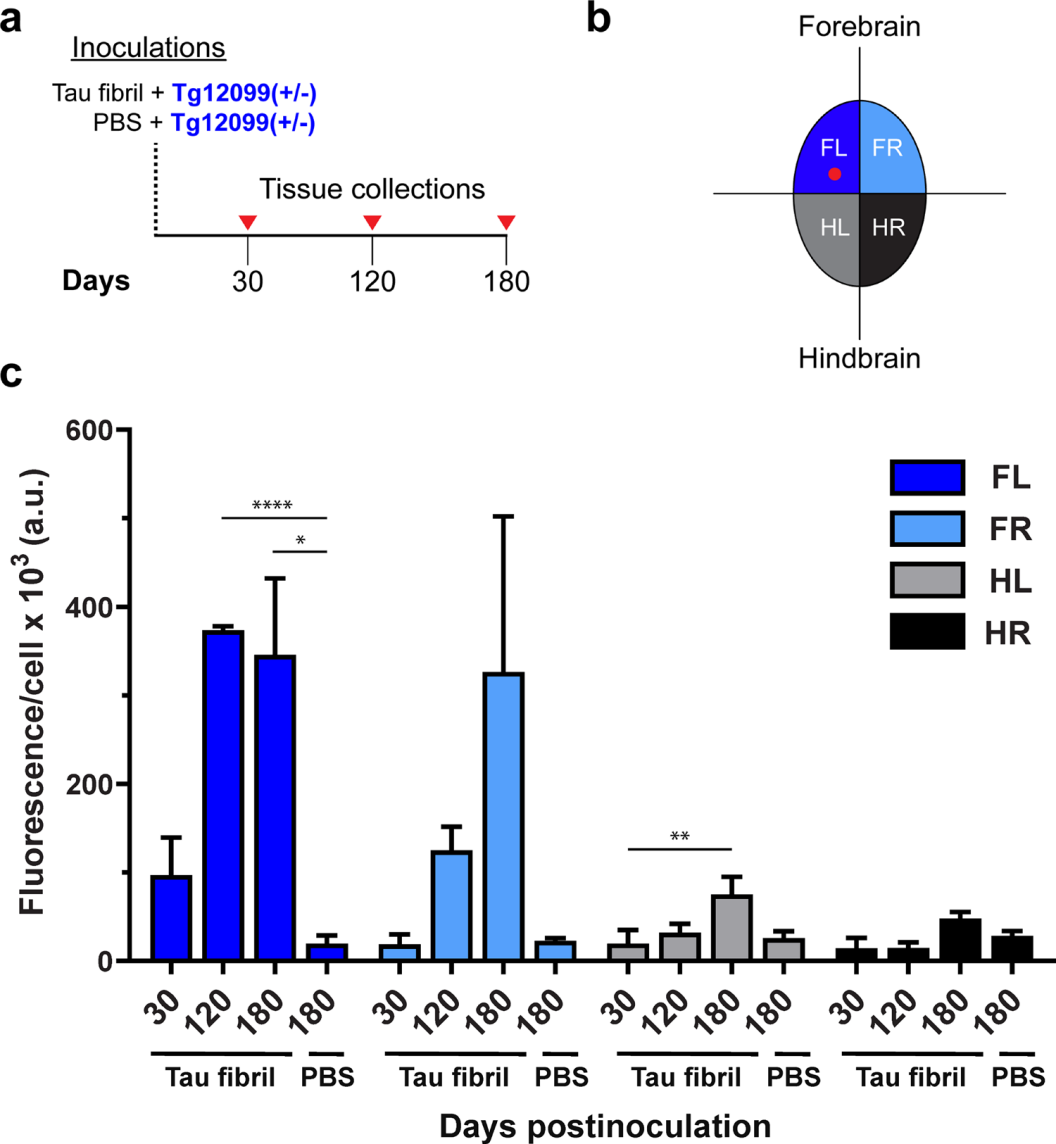
Rapid propagation and brain-wide spreading of induced tau prions in Tg12099(+/−) rats inoculated with recombinant tau fibrils. **a** Illustration of phosphate-buffered saline (PBS) injections or tau fibril injections into Tg12099(+/−) rats followed by tissue collections at 30-, 120-, and 180-days postinoculation (dpi). **b** Diagram of brain quadrants applied to HEK(tau-RD)-YFP cells to quantify tau prions. HL, hindbrain left (gray); HR, hindbrain right (black); FL, forebrain left (dark blue); FR, forebrain right (light blue). Red dot in FL is the location of the injection site. **c** Confocal microscopy measurements of fluorescence intensity of aggregates per cell reveals a rapid expansion of tau prions from the FL injection site into the other quadrants of Tg12099(+/−) rats. *a.u.* arbitrary unit. Data are represented as mean ± SEM (*n* = 3 rats per time point; *P < 0.05, **P < 0.01, ***P < 0.001, and ****P < 0.0001). Each time point per brain region was compared using a mixed-effects analysis followed by Tukey’s multiple comparisons test

Because seeding of tau fibrils led to global tau prion spread in Tg12099(+/−) rats (Fig. 8c), we wanted to measure this propagation via a cell-based assay. Since tau fibrils may induce a conformational change distinct from the tau prions observed in spontaneous tauopathy, we used HEK(tau-RD)-YFP cells, instead of HEK(P301S)-YFP cells, due to their increased sensitivity to infection with a variety of 4R tau prion strains [66, 78]. The brains of tau fibril–injected or PBS-injected Tg12099(+/−) rats were collected at 30-, 120-, and 180-days postinoculation (dpi) (Fig. 9a). Brains were dissected along the sagittal midline and then the forebrain and hindbrain halves were separated (Fig. 9b). These four brain regions were denoted as forebrain left (FL), forebrain right (FR), hindbrain left (HL), and hindbrain right (HR) (Fig. 9b). Lysates from homogenized brain quadrants were then applied to HEK(tau-RD)-YFP cells to measure fluorescence intensity of aggregates per cell. At 30 dpi, we found a rapid induction of tau prions in the FL quadrant (origin of the inoculation site) with no detectable spread in the remaining quadrants (Fig. 9c). At 120 dpi, the propagation of tau prions increased nearly 3.5-fold in the FL quadrant, with spread into the FR quadrant and to a lesser extent into the HL quadrant (Fig. 9c). While the propagation of tau prions reached a maximum at 120 dpi in FL (Fig. 9c), we continued to see the trend of increasing tau prion propagation in the FR quadrant from 120 to 180 dpi. Continued propagation of tau prions was found in the hindbrain quadrants at these later time points (Fig. 9c) yet did not lead to any observable phenotypes in the animals. PBS-injected Tg12099(+/−) rats at 180 dpi did not display any tau prion propagation (Fig. 9c), indicative of undetectable spontaneous disease in this genotype. Based on our neuropathological and cell-infectivity data, a single, unilateral intracerebral injection of recombinant tau fibrils led to a global, bilateral induction of tau prions in Tg12099(+/−) rats that progressed more rapidly than did spontaneous disease in Tg12099(+/+) rats.

### Transmission of brain-derived tau prions to young Tg12099(+/−) rats

We next asked if our tauopathy rat model would be susceptible to inoculation of brain-derived hTau prions. Using Tg12099(+/−) rats, we bilaterally injected tau prions into the thalamus from 1) the brains of aged, sick Tg12099(+/+) rats, 2) postmortem brain samples from human patients with clinically diagnosed AD and PSP, and 3) a postmortem brain sample from a cognitively unimpaired control human patient. Prior to inoculation, we increased the prion infectivity of rat and human brain samples by using limited proteolysis with PK followed by PTA precipitation (PK/PTA). This technique has been shown to increase the infectivity of human brain homogenates containing α-synuclein, tau, and Aβ prions in cell models of prion infection [6, 20]. These preparations resulted in significant infectivity when added to HEK(tau-RD)-YFP cells (Supplementary Fig. S9). Following inoculation at 6–8 weeks of age, Tg12099(+/−) rats were euthanized at various time points, and brains were hemi-dissected into forebrains and hindbrains. One hemisphere was used for pathological analysis by AT8 immunoreactivity, and the other was used to measure the abundance of tau prions using the HEK(tau-RD)-YFP cell model.

**Fig. 10:**
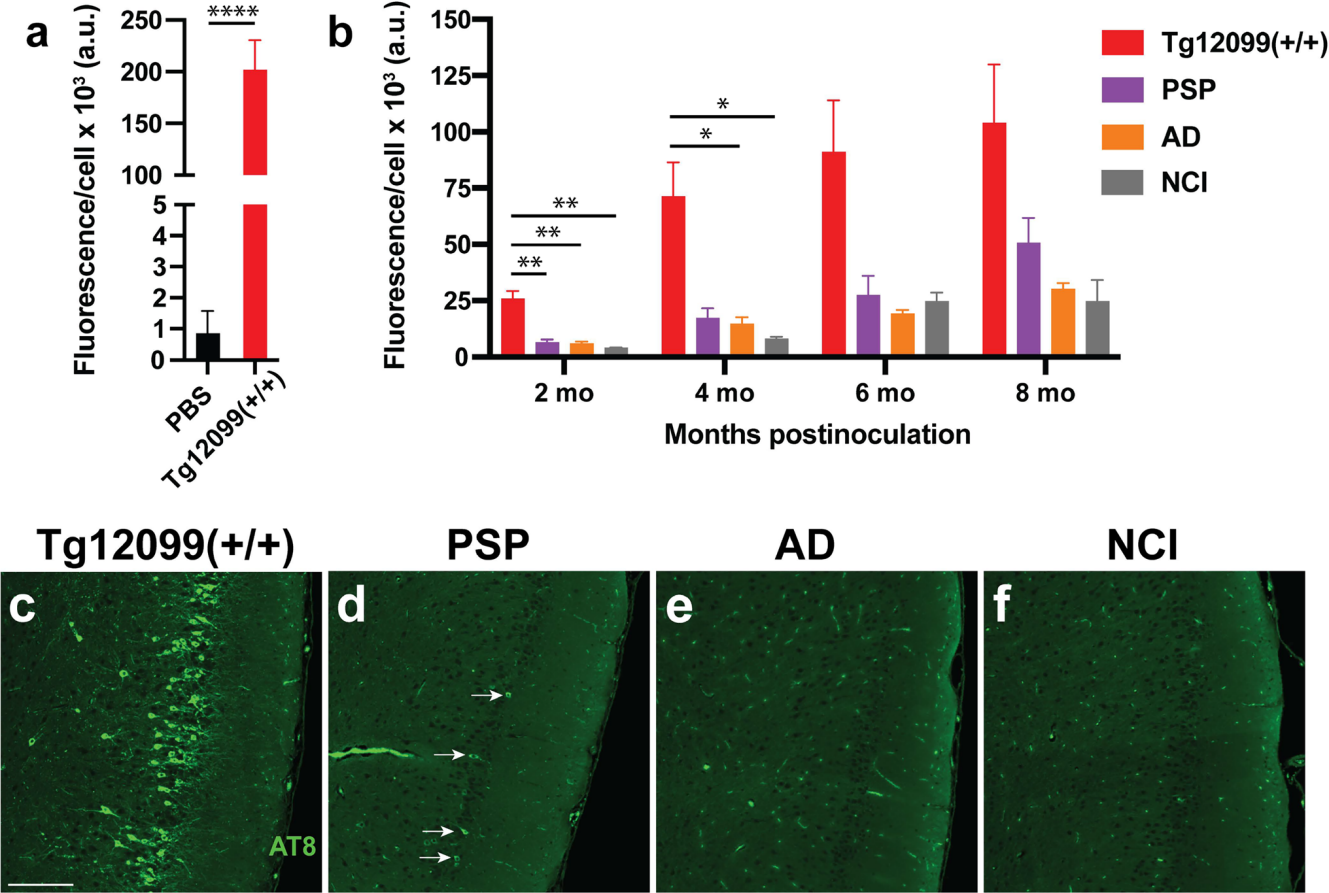
Efficient transmission of Tg12099(+/+) prions but not human PSP or AD prions to young Tg12099(+/−) rats. **a** A negative control (PBS) and positive control (homogenate from an aged Tg12099[+/+] rat) were included in each HEK(tau-RD)-YFP infectivity assay. **b** The accumulation of tau prions was measured in the brains of Tg12099(+/−) rats at various time points following the inoculation with rat or human patient samples, as indicated. AT8 immunoreactivity is shown in the entorhinal cortex from Tg12099(+/−) rats aged up to 8 months following inoculation with brain samples from **c** aged Tg12099(+/+) rats, **d** patients with progressive supranuclear palsy (PSP), **e** patients with Alzheimer’s disease (AD), and **f** cognitively unimpaired (NCI) patients. Scale bar = 100 µm. White arrows in (**d**) indicate AT8^+^ tau inclusions. Data are represented as mean ± SEM (*n* = 6 rats per age in months [mo]; *P* < 0.05, **P < 0.01, and ****P < 0.0001). Each time point per brain region was compared using a mixed-effects analysis followed by Tukey’s multiple comparisons test

First, we confirmed the infectivity of tau prions from aged Tg12099(+/+) rat forebrain homogenates by adding PK/PTA extracts directly to HEK(tau-RD)-YFP cells. This revealed a high level of prion activity, indicating an abundance of tau prions in the Tg12099(+/+) inocula (Fig. 10a). Following injection of Tg12099(+/−) rats with this preparation, we measured the level of tau prions in the forebrains at 2, 4, 6, and 8-months postinoculation (Fig. 10b, c). The abundance of tau prions increased over time (Fig. 10b), and immunofluorescence staining confirmed the presence of a significant AT8^+^ tau prion burden in the piriform cortex at 8-months postinoculation (Fig. 10c). Although tau prions were detected in the rats at these later timepoints, no discernible clinical phenotypes were observed.

PK/PTA preparations from brain homogenates of three separate AD and PSP cases were also investigated for propagation of tau prions in Tg12099(+/−) rats compared with a PK/PTA preparation from a brain homogenate of a cognitively unimpaired patient. Although rats inoculated with each of the three PSP patient samples appeared to accumulate a small amount of tau prions as measured in the HEK(tau-RD)-YFP cell model, the amount was not significantly elevated when compared with the infectivity from rats inoculated with the human control sample. However, PSP-inoculated rats did have infrequent AT8^+^ tau inclusions in the piriform cortex (Fig. 10d). Although we made PK/PTA preparations of each AD brain sample to increase the infectious titer (Supplementary Fig. S9), we did not measure prions above the levels detected in the negative control human sample in rats that were aged up to 8-months postinoculation (Fig. 10b). Furthermore, we did not detect any AT8^+^ immunoreactivity in the brains of rats inoculated with any of the AD patient samples or the cognitively unimpaired control patient sample (Fig. 10e, f).

## Discussion

Our studies demonstrate that, after tau proteins misfold into prions, the infectivity spreads through much of the CNS. Prions induce the misfolding of additional copies of the naïve protein (e.g., tau monomer) in a self-perpetuating process that spreads within and between neural cells (“infectivity” at the cellular level). Unlike conventional pathogens, prions lack a nucleic acid genome; their ability to propagate is instead enciphered in their three-dimensional conformation. The overexpression of MAPT*P301S tau allowed us to investigate the early onset of tau prion neuropathology in Tg12099 rats. The combination of this selective vulnerability, pTau accumulation, mature NFT formation, and focal neurodegeneration in Tg rats closely resembles the neuropathology and brain atrophy seen in humans with primary tauopathies. Thus, our study provides a link between the regional propagation of MAPT*P301S tau prions and neurodegeneration in vivo.

Recently, we demonstrated the utility of creating Tg rats for studying neurodegeneration and the propagation of PrP prions. We developed Tg2919 and Tg2922 rats overexpressing rat PrP at 4.4- and 9.7-fold higher levels, respectively, compared with WT animals [56]. Intracerebral injection of rat PrP prions into the brains of Tg(PrP) rats led not only to accelerated disease but also to selective propagation of PrP prions in the CNS; this result was similar to findings that showed that Tg mice can propagate human Creutzfeldt–Jakob disease (CJD) prions [73]. These findings suggest that, by using Tg rats in propagative models of neurodegeneration, we may be able to define regions of the rat CNS susceptible to other proteins that form prions, such as Aβ, α-synuclein, and tau.

The goal of the current study was to determine the regional vulnerability of the rat CNS to tau prions. We chose a pan-neural *RaPrnp* promoter to globally overexpress the MAPT*P301S mutation [13, 56, 71]. We posited that this Tg configuration would help us determine which regions in the rat brain are vulnerable to tau prions. Overexpression of MAPT*P301S in Tg12099(+/−) rats was ubiquitous in the CNS with certain regions expressing higher levels. We were surprised to see only a few AT8^+^ neurons in the entorhinal cortex of 18-month-old heterozygous rats. Recent studies by others have demonstrated that misfolded hTau may be degraded by protein-clearance mechanisms [16, 38, 75]. To overcome potential processes of hTau clearance in the Tg12099(+/−) rat, we increased the dosage of hTau by producing homozygous Tg12099(+/+) rats, which are prone to neurological decline and death. This phenotype could have been caused by the transgene inserting into a gene, with a recessive mutation becoming penetrant on the Tg12099(+/+) background. To address this possibility, we performed TLA but did not observe the RaPrnp-MAPT*P301S transgene integrating near or into a genetic locus on rat chromosome 18. Thus, the tauopathy phenotypes described here arose from the MAPT*P301S transgene.

Previous studies have capitalized on the self-templating and aggregating nature of misfolded tau by using a fluorescent-tagged protein in HEK cells to measure the levels of tau prions [47, 49, 66, 78, 79]. We obtained similar results using cell-based assays for tau prion accumulation. We found tau prions increased in a time-dependent manner in cortical and limbic structures concurrently, as measured by immunostaining with the monoclonal antibodies AT8 and MC1 in Tg12099(+/+) rats. In addition, we found similar increases in tau prions, as detected by the amyloid dye ThioS and Bielschowsky silver staining.

In contrast to the tau deposition found in the cortical and limbic structures of Tg12099(+/+) rats, the brainstem and mts displayed less staining using these tauopathy markers. Notably, the cortical and limbic structures had a significant tau prion burden in the cell assay as early as 9 months of age. We suspect that tau prions in these regions may have a conformation sufficient for templating the MAPT*P301S-YFP fusion protein but do not acquire hyperphosphorylation or conformational changes as shown with AT8, MC1, ThioS, and Bielschowsky silver staining methods. Our findings are consistent with prior results using human brain samples in which robust tau prion abundance was found. Importantly, however, these human brain samples lacked overt histological staining of pathologic tau [29, 48]. The cortex and hippocampus of Tg12099(+/+) rats had the highest tau prion burdens in addition to prominent neurodegeneration and gliosis, which resembles disease phenotypes seen in patients with FTLD-tau [8]. Thus, our Tg12099 rat model may prove useful in modeling human tauopathies. Intriguingly, the cerebellum appeared to be resistant to the propagation of tau prions and did not stain for tauopathy markers in Tg12099(+/+) rats.

A small number of Tg tau mouse models have been reported to accumulate tau pathology following inoculation with brain samples from human patients with tauopathy [17, 31, 40, 65]. A characteristic of these models that makes them ideal candidates as permissive hosts for tau prion accumulation is that they do not accumulate high pathological tau burdens throughout their lifespans. Seeing as this trait was also observed in the Tg12099(+/−) rats, we tested their permissiveness by infecting them with several tau prion–containing inocula, including recombinant fibrils, brain samples from aged Tg12099(+/+) rats, and brain samples from human tauopathy patients. We detected a robust accumulation of tau prions in the forebrains of Tg12099(+/−) rats inoculated with recombinant K18-P301L fibrils at only 2-months postinoculation. PK/PTA preparations of brains from aged and diseased Tg12099(+/+) rats were inoculated into Tg12099(+/−) rats and were also found to induce measurable quantities of tau prions as early as 2-months postinoculation. The tau prions increased in abundance up to 8-months postinoculation when we collected the last cohorts. Although we observed several AT8^+^ neurons within the piriform cortex of PSP-inoculated rats, we did not detect a significant increase of tau prions measured in the HEK(tau-RD)-YFP cell model with any of the three PSP or three AD human brain samples we inoculated, when compared with the cognitively unimpaired control human brain sample. The titers of tau prions in human patient samples subjected to PK digestion and PTA precipitation were insufficient to permit studies in the Tg12099(+/−) rats when aged to 8-months postinoculation.

While a Tg rat expressing the repeat domain of hTau has been developed by others [85], the Tg12099 rat expresses the full-length hTau 0N4R isoform containing a familial mutation that may be better suited for modeling human tauopathy. This is consistent with the recent report of human-like tauopathy in a different Tg rat model expressing the hTau 2N4R isoform under the forebrain-restricted Camk2A promoter [24]. Given that the brains of rats are approximately three times larger than mice, the use of new tau PET ligands in Tg12099 rats might provide an excellent opportunity for noninvasive longitudinal imaging. Based on our observations showing intense PBB3 labeling of tau tangles in ex vivo slices, the Tg12099(+/+) rat may be a suitable preclinical model to test tau-binding PET ligands to diagnose disease, monitor progression, and assess therapeutic efficacy. Furthermore, the extensive behavioral repertoire of rats makes the Tg12099 model ideal for studying how pathogenic tau load correlates with behavioral phenotypes. Lastly, the larger size of rats makes sequential collections of cerebrospinal fluid and plasma easier. This will be important for determining if key biomarkers associated with AD and other tauopathies are present in Tg12099 rats and if they can be used to noninvasively monitor disease progression and therapeutic intervention.

## Conclusions

Homozygous Tg12099(+/+) rats develop the pathological hallmarks of tauopathies that include severe atrophy in the piriform cortex and amygdala. In contrast, the hemizygous Tg12099(+/−) rats did not spontaneously develop pathological tau prion accumulation. Exogenous administration of tau prions induced disease in this hemizygous line. These highly reproducible and significant changes might provide an opportunity to discover therapeutics that can halt progression of human tauopathies, including AD.

### Supplementary Information

Below is the link to the electronic supplementary material.

Supplementary file1 (PDF 1411 KB)

## Data Availability

TLA fastq files were deposited at the NCBI Sequence Read Archive under BioProject accession PRJNA544101 (http://www.ncbi.nlm.nih.gov/bioproject/PRJNA544101).
