# Supplementary material for: Severe neurodegeneration in brains of transgenic rats producing human tau prions
Source: Acta Neuropathol. 2024 Aug 20;148(1):25. doi: 10.1007/s00401-024-02771-5 (PMC11333523; doi:10.1007/s00401-024-02771-5)
Supplement: Supplementary file 1 — Supplementary file1 (PDF 1411 KB) [file 401_2024_2771_MOESM1_ESM.pdf]

SUPPPLENTARY INFORMATION

Severe neurodegeneration in brains of transgenic rats producing human tau prions

Jacob Ayers,[1,2†] T. Peter Lopez,[1†] Ian T. Steele,[1] Abby Oehler,[1] Rigo Roman-Albarran,[1] Elisa Cleveland,[1] Alex Chong,[1] George A. Carlson,[1,2] Carlo Condello,[1,2*] and Stanley B. Prusiner[1,2,3*]

[1]Institute for Neurodegenerative Diseases, Weill Institute for Neurosciences, University of California, San Francisco, CA 94158; [2]Department of Neurology, Weill Institute for Neurosciences, University of California, San Francisco, CA 94158; [3]Department of Biochemistry and Biophysics, University of California, San Francisco, CA 94158

***Correspondence:** carlo.condello@ucsf.edu, 415-502-7075; stanley.prusiner@ucsf.edu, 415-476-4482

[†]Jacob Ayers and T. Peter Lopez contributed equally to this work.

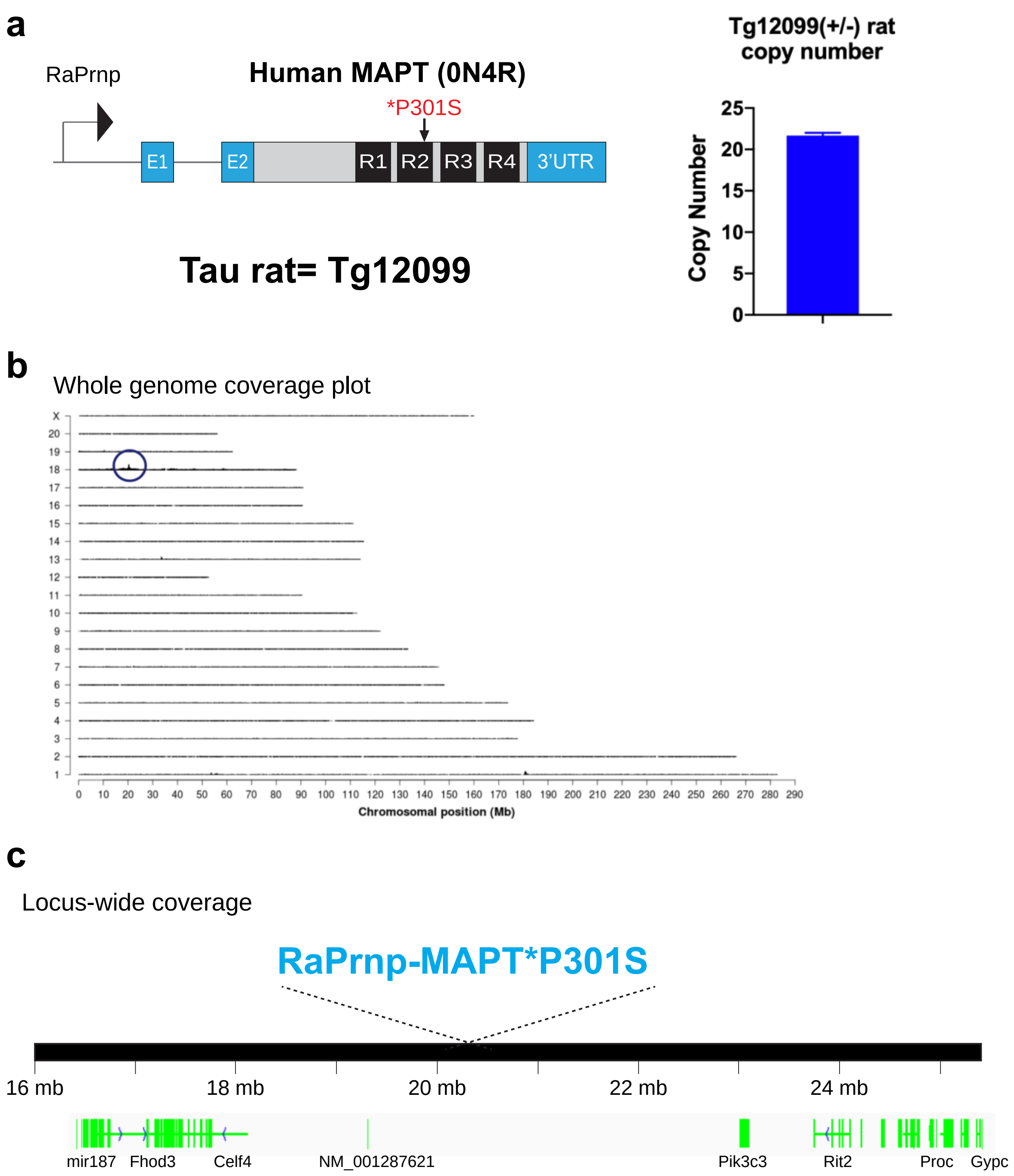

Fig. S1 Generation of Tg12099 rats to model human tauopathy

(a) Schematic describing the approach used to generate transgenic (Tg) rats overexpressing MAPT*P301S. A plasmid construct was created to contain the rat prion promoter (*RaPrnp*)

upstream of a human tau 0N4R isoform with the MAPT*P301S mutation. The construct was linearized and microinjected into one-cell-stage rat zygotes, which were then transferred to a pseudopregnant female that birthed the Tg12099 rat line. Tg12099(+/-) rats (n = 3) were found to have 21 to 22 copies of the transgene by droplet digital polymerase chain reaction analysis. (b) Targeted locus amplification (TLA) revealed the *RaPrnp-MAPT*P301S* transgene had a single insertion site located in rat chromosome 18:20,312,421. (c) Magnification of the TLA sequencing reads shows that the transgene did not insert near or into a genetic locus. Mb, megabase.

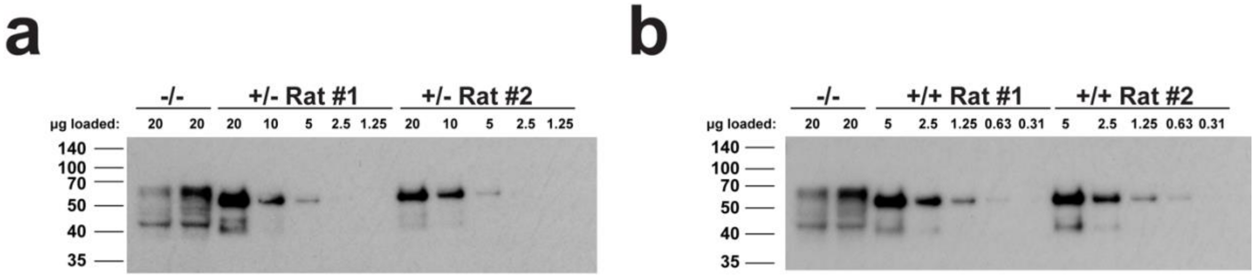

Fig. S2 Approximate protein levels of human tau overexpression in Tg12099 rats

Various concentrations of total protein from brain homogenates were immunoblotted using the Tau5 antibody. Densitometry of signals from each lane was determined, and the concentration of brain homogenate from (a) Tg12099(+/-) and (b) Tg12099(+/+) rats that gave a similar intensity to 20 µg of brain homogenate from a WT Sprague Dawley rat (-/-) was approximated. Brains from two Tg12099(+/-) rats and two Tg12099(+/+) rats were used and are shown above.

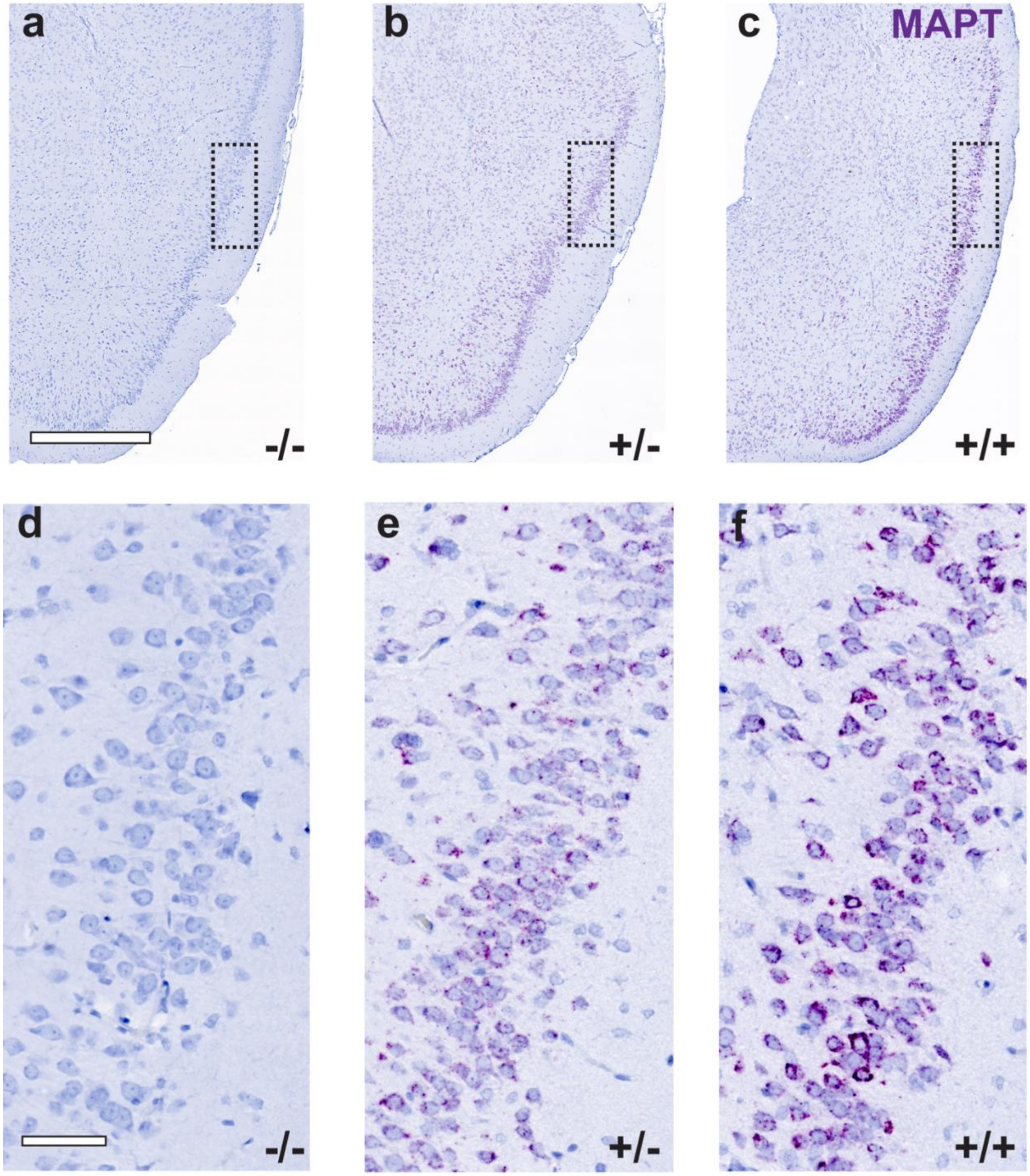

Fig. S3 In situ hybridization for human-specific *MAPT* shows gene dose–dependent expression of RNA transcripts predominantly in neurons

(a–c) Low magnification view of piriform cortex region in fixed coronal sections from aged (a) wild-type (-/-), (b) Tg12099(+/-), and (c) Tg12099(+/+) rats. Scale bar = 1 mm. (d–f) Insets correspond to dotted outlines in panels a–c show magnified views of the piriform cortex and gene dose dependence of $MAPT^{+}$ cells. Scale bar = 50 µm. A human *MAPT*–specific probe labels single RNA transcripts of *MAPT* (magenta). Sections are counterstained with hematoxylin to show cellular morphology (blue).

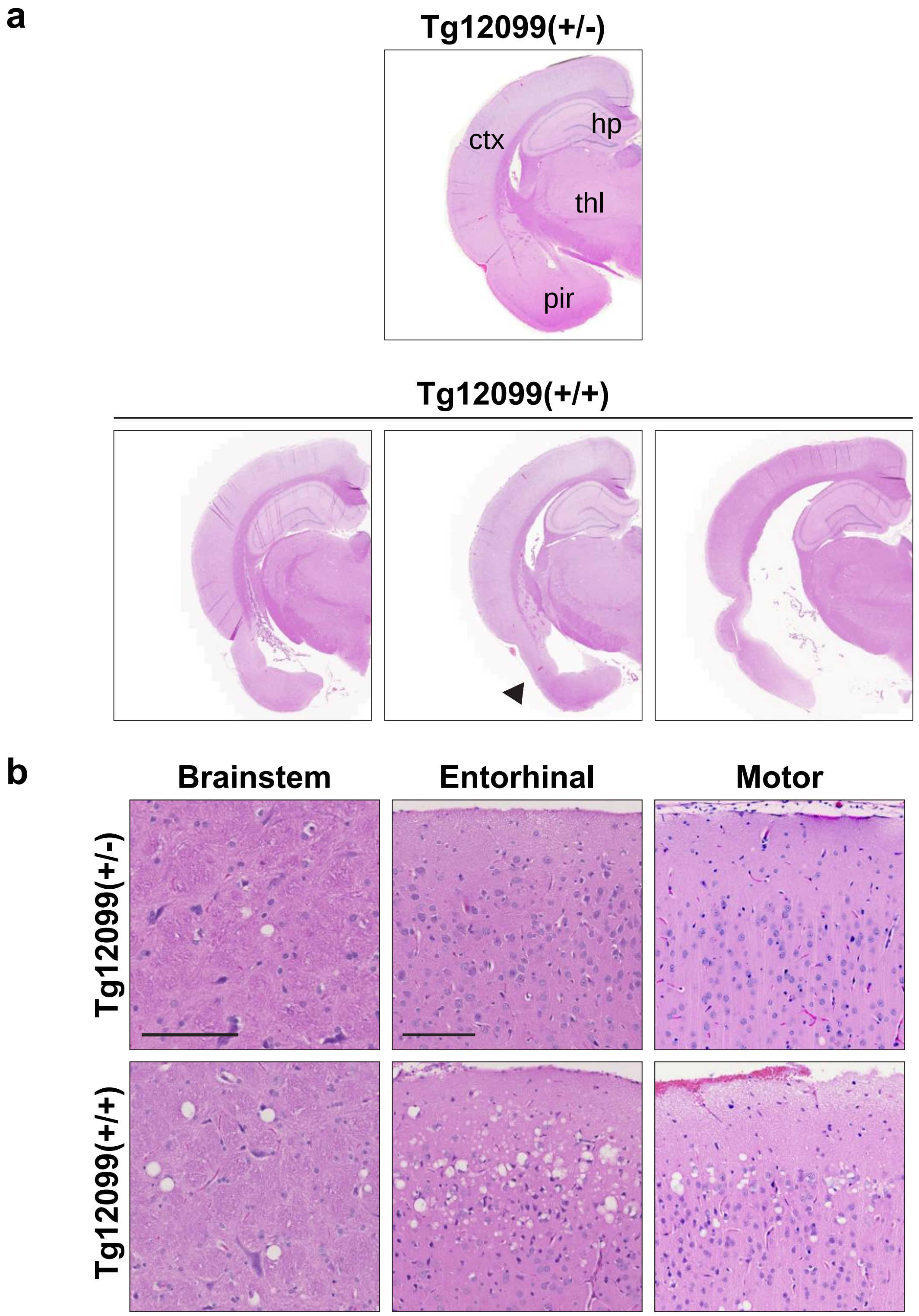

Fig. S4 Neurodegeneration in Tg12099(+/+) rats

(a) Low magnification of coronal hematoxylin and eosin (H&E)–stained brain sections from a 14-month-old Tg12099(+/-) rat (top image) compared with terminal Tg12099(+/+) rats (bottom images). Arrowheads in images of Tg12099(+/+) rat brain sections point to selective vulnerability of the entorhinal and piriform cortices to neurodegeneration. (b) Normal H&E pathology in Tg12099(+/-) rats (top panels) compared with vacuolization in the brainstem and entorhinal and motor cortices in Tg12099(+/+) rats (bottom panels). All scale bars = 100 μm. Scale bar in the entorhinal cortex also applies to the images of the motor cortex.

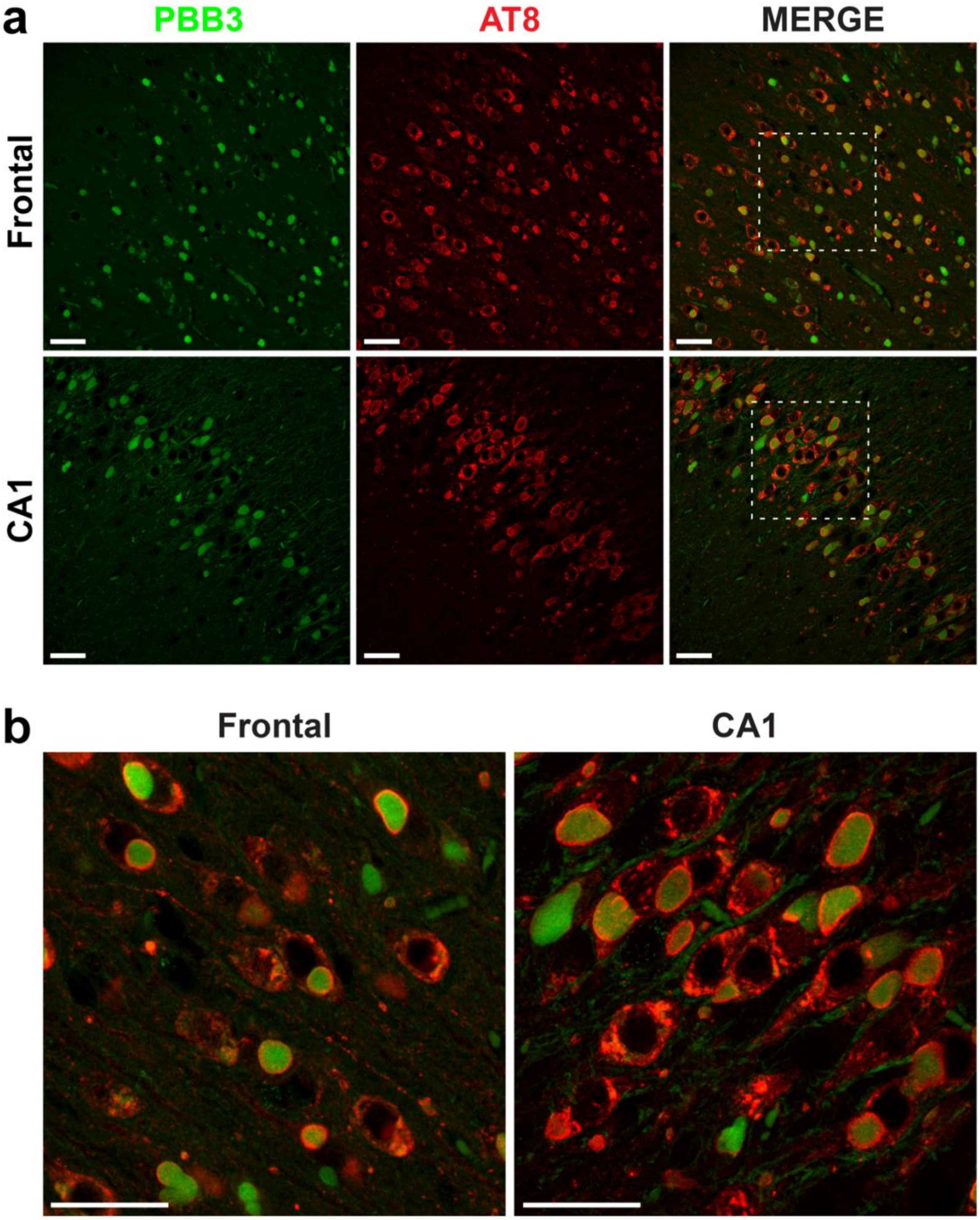

Fig. S5 AT8+ inclusions colocalize with the PBB3 tau positron emission tomography ligand

(a) Low magnification of the frontal cortex (top panels) and CA1 layer (bottom panels) stained for PBB3 (green), AT8 (red), and merged into one image. Scale bar = 50 µm. Dotted squares denote magnified regions in (b). Scale bar = 25 µm.

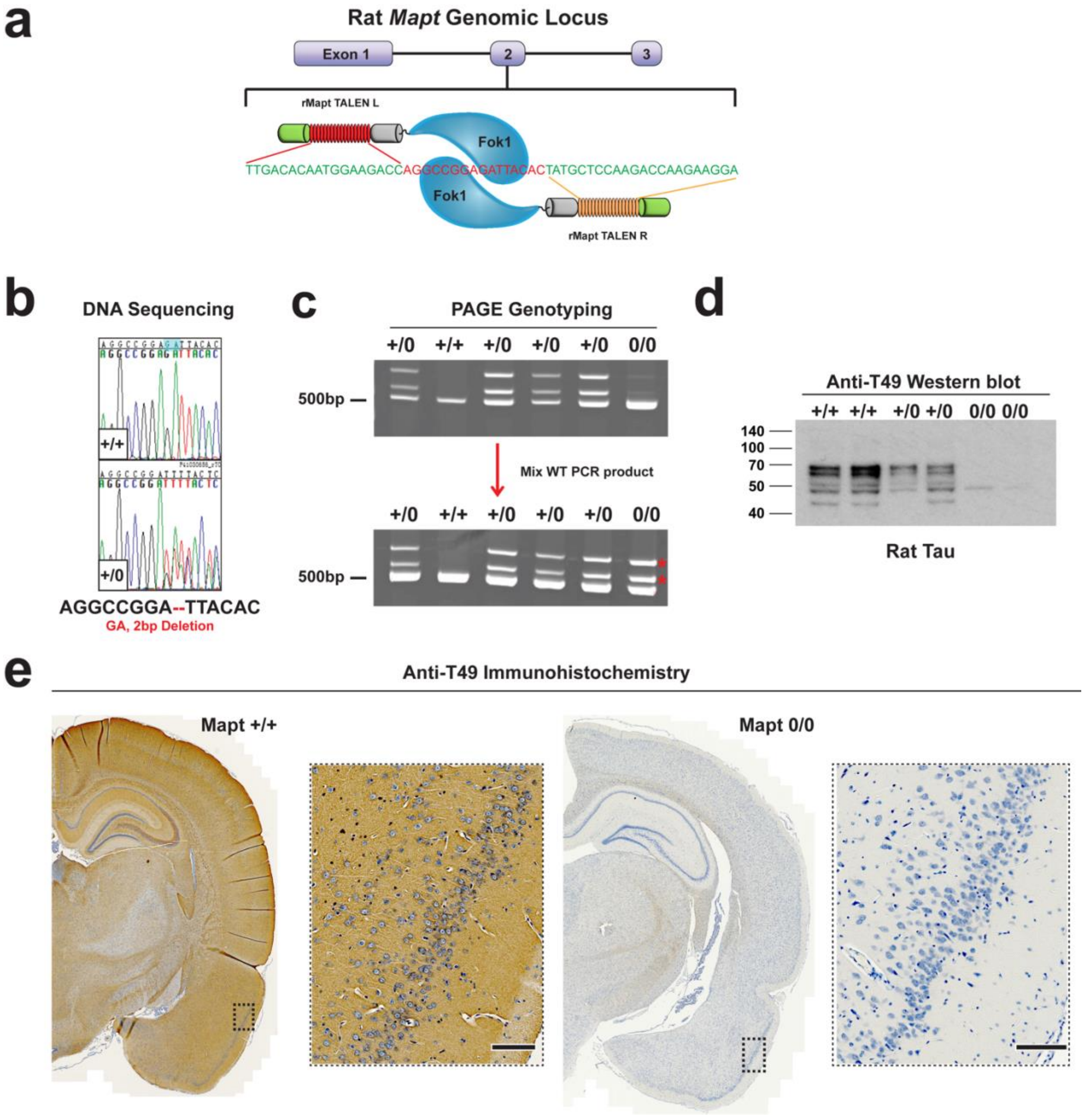

Fig. S6 Generation and molecular phenotyping of Mapt knockout rats

(a) Schematic of Transcription activator-like effector nuclease (TALENs) targeting Exon 2 of the rat *Mapt* genomic locus. Two TALENs were designed, rMapt (L) and rMapt (R), to flank 5’ and 3’ sequences (green) respectively of Exon 2 to allow for double-strand breaks (red) by the Fok1

nuclease. (b) DNA sequencing demonstrates a 2-bp deletion (GA) in a heterozygous (+/0) rat (bottom chromatogram) to a wild-type (WT) rat (top chromatogram). (c) To screen the F2 generation for homozygous (0/0) mutants, we mixed WT (+/+) PCR products with denatured products isolated from F2 littermates (top PAGE gel) to reveal four heterozygous rats (lanes 1, 3-5), one WT rat (lane 2), and one homozygous mutant rat, referred to as the Cy23 line (bottom PAGE gel, lane 6). Red asterisks mark DNA heteroduplexes where the rehybridized GA deletion in the mutant are mismatched to WT DNA to create an open angle that migrates at a slower pace.(d) To determine if the tau knockout rat is null for producing tau protein, we performed Western blot analysis using T49 antibody to stain endogenous rat tau in brain homogenates from 6-month-old WT (n = 2), Mapt +/0 (n = 2), and Mapt 0/0 (n = 2) littermates. (e) Brightfield images of T49-immunolabeled coronal brain slices from WT and Mapt 0/0 rats. All scale bars = 100 μm.

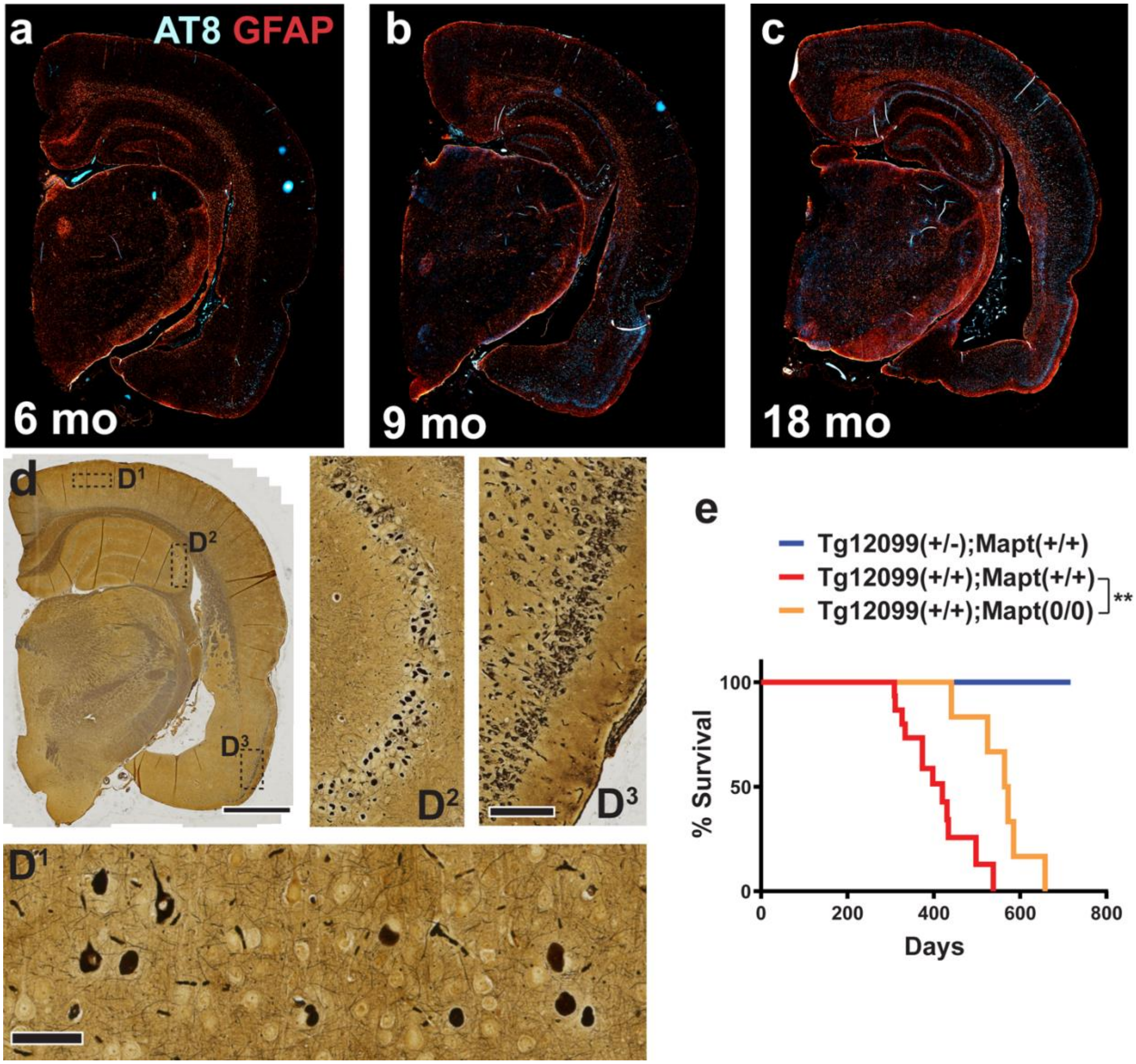

Fig. S7 Rat *Mapt* knockout does not affect tau pathology phenotype in Tg12099+/+ rats but does extend survival

(a–c) Low magnification view of coronal brain sections from Tg12099(+/+) rats at 6, 9, and 18 months of age immunostained with AT8 (cyan) and GFAP (red). (d) Bielschowsky silver staining demonstrates widespread mature tau tangle pathology in the superficial sensory cortex (D^1), the CA3 region of the hippocampus (D^2), and the piriform cortex (D^3). Scale bars: d = 2 mm, D1 =

25 µm, and D2/D3 = 100 µm. (e) Kaplan–Meier plot demonstrates survival percentage of Tg12099(+/-);*Mapt*+/+ rats (n = 4), Tg12099(+/+);*Mapt*+/+ rats (n = 13), and Tg12099(+/+);*Mapt*(0/0) rats (n = 8). We used a log-rank Mantel–Cox test to compare Tg12099(+/+);*Mapt*+/+ rats and Tg12099(+/+);*Mapt*(0/0) rats. ***P* = 0.0013. mo, months.

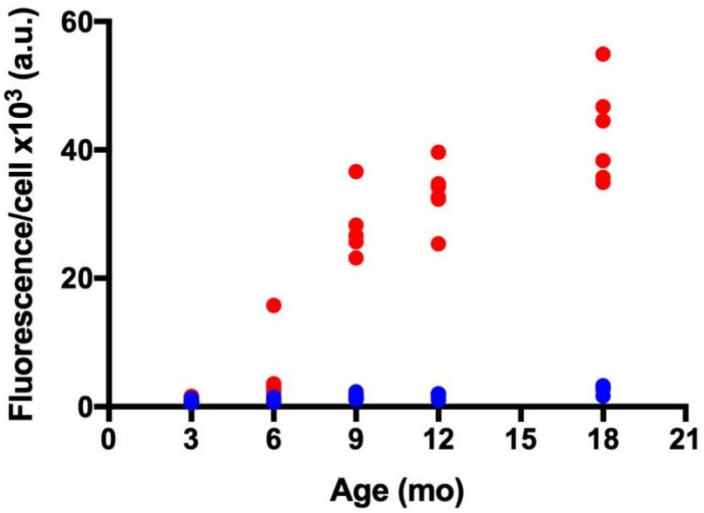

Fig. S8 Self-propagation of tau prions in Tg12099 rat brains

Using the tau prion cellular assay, whole brain lysates from Tg12099(+/+) rats had increasing tau prion levels over time compared with lysates from Tg12099(+/-) rats. Blue circles, individual Tg12099(+/-) rats; red circles, individual Tg12099(+/+) rats. n = 3–6 rats per age in months (mo). a.u., arbitrary unit.

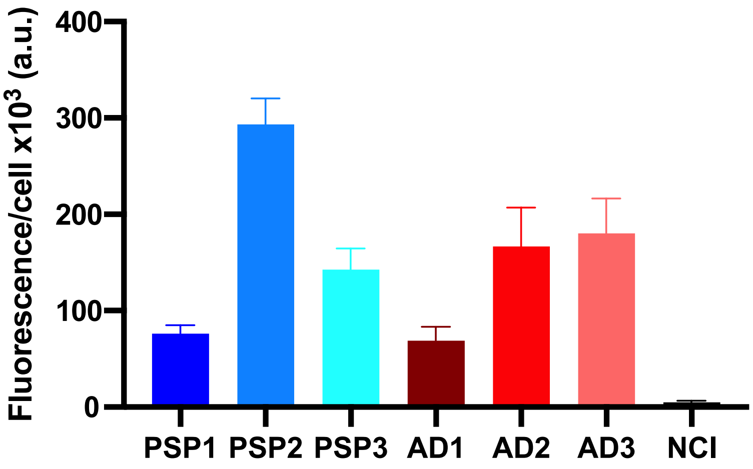

Fig. S9 Proteinase K digested/phosphotungstic acid precipitated (PK/PTA) samples isolated from the brains of human patients with progressive supranuclear palsy (PSP) or Alzheimer's disease (AD) are infectious in cellular assay for tau prions

Brain samples from three patients with PSP and three patients with AD were processed by PK/PTA precipitation and shown to be infectious in HEK293T cells transfected with a yellow fluorescent protein (YFP) fusion construct expressing truncated human tau, HEK(tau-4R repeat domain*P301L/V337M)-YFP. The control, a cognitively unimpaired (NCI) patient brain sample, was also processed by PK/PTA precipitation and showed no infectivity in the same cell model. Data are represented as mean ± SEM (n = 6 technical replicates).

Table S1. PCR primers and ampified products used in this study.

PCR amplified product	Forward primer	Reverse primer
In-Fusion fragment: 15 bp RaPrnp H. arm/Human MAPT ORF/15 bp RaPrnp H. arm	5'-TAGCGGTACCCTCGAG CCGCCACCATGGCTGAG-3'	5'-AACAAGCAGGCTCGAT CACAAACCCTGCTTGGC-3'
MAPT*P301S: site-directed mutagenesis primers. **Bold:** P301S substitution	5'-AATATCAAACACGTC**TCG**G GAGGCGGCAGTG-3'	5'-CACTGCCGCCTCC**CGA**GA CGTGTTTGATATT-3'
RaPrnp promoter TLA sequencing primers	5'-CCACTCATTTCTAACAGGCT-3'	5'-CCTACCTGGTTATTATT GACATG-3'
MAPT*P301S TLA sequencing primers	5'-GTGGCAGTGGTCCGTACT-3'	5'-ATCGCTTCCAGTCCCGTC-3'
RaMAPT*P301S genotyping primer	5'-CAGCATCGACATGGTA GAC-3'	5'-CCACGAGAATGAGAAG GAAC-3'
rMapt Exon 2 Sequencing primers	5’-TCATATCGCCAGGGTCAGCATC-3’	5’-CCCTGCTGAGGCAAACACAGACT-3’
PAGE rMapt Exon 2 genotyping primers	5'-TCCCTCCATAACCACTGGAC-3'	5'-TGCTGAGGCAAACACAGAC-3'

Abbreviations: bp, base pair; ORF, open reading frame; PCR, polymerase chain reaction; TLA, targeted locus amplification.

Table S2. Antibodies used in this study.

Antibody	Species	Dilution	Source
Tau13 (human-specific)	Mouse	WB: 1:5,000<br>Path: 1:1,000	Produced in-house
Tau5	Mouse	WB: 1:1,000	Produced in-house
T49 (rodent-specific)	Mouse	WB: 1:1,000<br>Path: 1:1,000	Millipore (#MABN827)
AT8	Mouse	WB: 1:1,000<br>Path: 1:250	Thermo Fisher Scientific
PHF-1	Mouse	WB: 1:1,000	Gift from Peter Davies (Feinstein Institutes for Medical Research, Manhasset, NY)
MC1	Mouse	Path: 1:400	Gift from Peter Davies (Feinstein Institutes for Medical Research, Manhasset, NY)
NeuN	Guinea Pig	Path: 1:200	Synaptic Systems (#266 004)
Gfap	Chicken	Path: 1:500	Abcam (#ab4674)
Iba1	Rabbit	Path: 1:500	Abcam (#ab178846)
Anti-mouse–HRP conjugated	Goat	WB: 1:5,000–1:10,000	Thermo Fisher Scientific
Alexa Fluor–conjugated anti-species IgG	Goat	Path: 1:500	Thermo Fisher Scientific

Abbreviations: IgG, immunoglobulin; HRP, horseradish peroxidase; Path, pathology; WB, Western blot.

Table S3. Numbers of Tg12099(+/-) rats used for inoculations.

Inoculum	No. of Tg12099(+/-) rats collected at each time point			
	2 months	4 months	6 months	8 months
Aged Tg12099(+/+) PK/PTA brain prep	6	6	6	6
PSP patient PK/PTA brain prep				
Patient #1 (P2496)	5	6	6	6
Patient #2 (P2326.1)	5	6	6	6
Patient #3 (P2637)	5	6	6	6
AD patient PK/PTA brain prep				
Patient #1 (ADSwe#1)	5	5	6	6
Patient #2 (ADSpor#10)	5	5	6	6
Patient #3 (2312.1)	3	3	4	4
Negative control patient PK/PTA brain prep	5	6	6	6

Abbreviations: AD, Alzheimer's disease; PK/PTA, proteinase K digested/phosphotungstic acid precipitated; PSP, progressive supranuclear palsy.
